# Bayesian and non-Bayesian estimation of the bivariate inverse Weibull distribution parameters using ranked set sampling with concomitant variable

**DOI:** 10.1038/s41598-025-23741-1

**Published:** 2025-11-07

**Authors:** Hiba Z. Muhammed, Mostafa Shaaban

**Affiliations:** 1 Faculty of Graduate Studies for Statistical Research, Cairo University, Giza, Egypt; 2 Department of Statistics, Giza Higher Institute for Managerial Sciences, Giza, Egypt

**Keywords:** Bivariate inverse weibull distribution, Ranked set sampling, Maximum likelihood estimation, Bayesian estimation, Marshall-Olkin bivariate models, Engineering, Mathematics and computing

## Abstract

Estimating the bivariate distribution parameter is crucial for modeling paired variable dependencies, but highly variable or resource-intensive data may not respond well to traditional simple random sampling (SRS). In order to maximize efficiency, Ranked Set Sampling (RSS) ranks a subset of observations based on a concurrent variable, hence selecting just a subset for measurement. This study use both Bayesian and non-Bayesian estimation techniques to estimate the parameters of the Bivariate Inverse Weibull (BIW) distribution under RSS and SRS. According to the Marshall-Olkin approach, dependencies are captured by the BIW model using the parameters. We compute the probability functions for RSS and SRS because the ranking technique and dependence structure are intricate. Based on SRS and RSS, Bayesian estimators are explicitly derived by applying conjugate gamma priors for model parameters under squared error loss, whereas Maximum Likelihood Estimation (MLE) solutions are derived numerically via the Newton-Raphson technique because of the likelihood equations’ nonlinearity. Mean Squared Error (MSE), Bias, and Efficiency (EFF), simulations conducted with four different parameter settings that showed that RSS routinely performs better than SRS. In particular, under RSS, Bayesian estimation frequently produces lower MSE and bias than MLE. Nevertheless, prior decisions have an impact on Bayesian performance, particularly when the parameters are tiny, Simulations with 10,000 Monte Carlo replications across four parameter sets show that RSS consistently outperforms SRS, with MSE reduced by up to 50% and EFF exceeding 10 for large samples. Bayesian estimation with conjugate gamma priors yields lower MSE than MLE, particularly under RSS, though prior selection is critical for small parameters. We recommend RSS with Bayesian methods for applications in reliability and lifespan analysis, as demonstrated on a real dataset of 243 men’s body fat and chest circumference.

## Introduction

Parameter estimation is a fundamental aspect of statistical modeling, particularly for complex data structures such as bivariate distributions that capture dependencies between paired variables. Traditional estimation methods often rely on Simple Random Sampling (SRS), which assumes observations are independently and identically distributed. However, SRS can be inefficient when data collection is resource-intensive or when the population exhibits high variability. Ranked Set Sampling (RSS), proposed by Ref^1^. , addresses these limitations by incorporating auxiliary information to rank observations without requiring full measurement. RSS selects subsets of units, ranks them based on a concomitant variable, and measures only a subset of ranked units, thereby achieving greater statistical efficiency than SRS for the same sample size^2^.

RSS has proven valuable across diverse fields, including environmental science, agriculture, and reliability engineering, where ranking based on auxiliary or concomitant variables enhances data collection efficiency^3^. For bivariate distributions, RSS is particularly effective as it leverages the dependence between paired variables by using one variable to rank the other, thus improving the estimation of joint distributional properties.

Estimating parameters for bivariate distributions under RSS presents challenges due to the complex likelihood functions arising from the ranking mechanism and the dependence structure. However, the resulting equations are often non-linear, necessitating numerical methods like the Newton-Raphson algorithm. In contrast, Bayesian estimation incorporates prior knowledge through conjugate priors, producing posterior distributions that can be derived analytically or numerically under loss functions such as squared error loss. Bayesian methods are particularly advantageous when prior information is available or when sample sizes are small, offering robust uncertainty quantification.

The application of RSS to bivariate distributions, particularly the BIW model, is an emerging area with significant practical relevance. For^4^ developed RSS methodologies using concomitant variables for paired data.

The bivariate approach encourages researchers to choose a suitable distribution for the paired variables (X, Y). For instance, Ref.^5^ suggested using an auxiliary variable X to rank the units and the mean of an RSS to estimate the mean of the target variable Y within the context of the bivariate normal distribution. Reference^6^ on the other hand, used RSS to create the best linear unbiased estimator (BLUE) for the mean of Y. Over the years, several RSS variations have been introduced. Reference^7^ proposed a modified RSS method that selects either the smallest or largest ranked unit from each set for analysis, later known as Lower RSS (LRSS) or Upper RSS (URSS). Reference^8^ examined extreme RSS (ERSS) techniques for estimating population means. Additional RSS variants, including Median RSS (MRSS) and Moving Extreme RSS (MERSS), have been explored by Refs^9,10^.

RSS was used by Ref^11^. to estimate a two-parameter exponential distribution’s parameters. Reference^12^ formulated the BLUE for a parameter of the study variable in a Morgenstern-type bivariate exponential (MTBE) distribution, using both RSS and censored RSS approaches. Reference^13^ made inferences about Downton’s bivariate exponential distribution using moving extreme RSS. Reference^14^ also estimated parameters of Downton’s bivariate exponential distribution through RSS. The Morgenstern-type bivariate generalized exponential distribution was examined by Ref.^15^, who also proposed estimators for the population mean across several RSS schemes. Reference^16^ proposed an ordered extreme RSS method and applied it to estimate parameters for the Morgenstern type bivariate exponential distribution, while Ref.^17^. introduced a Bayesian estimator for the study variable’s mean in the same distribution. Reference^18^ developed estimators for the scale parameter of the study variable in a Cambanis-type bivariate uniform distribution using diverse RSS methods. Reference^19^ recently proposed parameter estimation for the Farlie–Gumbel–Morgenstern bivariate Bilal distribution under a multistage RSS framework. For more details see Refs.^20–23^.

The novelty lies in deriving the likelihood for Marshall-Olkin bivariate models under RSS for the first time and comparing Bayesian vs. Maximum Likelihood Estimation (MLE) performance via simulations.

## Description of the data and model assumption

### Bivariate inverse Weibull model

The Inverse Weibull (IW) distribution is a well-established model in reliability and survival analysis, known for its ability to capture non-monotone hazard functions, making it particularly useful for modeling lifetime data with heavy-tailed characteristics. In recent years, the extension of the IW distribution to the bivariate case has garnered significant attention due to its applicability in modeling dependent random variables, such as paired lifetimes in engineering, medical, and economic contexts. The IW distribution, first introduced for modeling degradation phenomena in mechanical components, such as diesel engines, is characterized by its flexibility in handling non-monotone hazard rates, which can be decreasing, increasing, or upside-down bathtub-shaped. The BIW distribution extends this framework to model two dependent random variables, ensuring that the marginal distributions remain IW. This is particularly valuable in reliability and survival analysis, where paired lifetimes (e.g., failure times of two components in a system) exhibit dependence. A seminal contribution to the BIW distribution is the work by Ref.^24^, who introduced a BIW model analogous to the Marshall–Olkin bivariate exponential distribution. Their formulation ensures that the marginals are IW distributions and includes a singular component to account for simultaneous failures, a feature common in reliability contexts where components may fail together due to a common shock. Reference^25^ provides a detailed construction of the Marshall–Olkin type BIW distribution, which is built using three independent IW random variables, assume three independent random variables, $\:{U}_{1},{U}_{2}$ and $\:{U}_{3}$, $\:i=\text{1,2},3$ and let $\:{U}_{i}$~ IW $\:\left({\alpha\:,\:\lambda\:}_{i}\right)$, IW distribution with shape and scale parameters (α, $\:\lambda\:)$ are indicated by IW (α, $\:\lambda\:$). The inverted Weibull random variable’s cdf and pdf, are

1 $$\:{F}_{IW\:}\left(x;\alpha\:,\:\lambda\:\right)={e}^{-\lambda\:{x}^{-\alpha\:\:}},$$

2 $$\:{f}_{IW\:}\left(x;\alpha\:,\:\lambda\:\right)=\alpha\:\:\lambda\:\:{x}^{-\alpha\:-1\:}{e}^{-\lambda\:{x}^{-\alpha\:\:\:}\:},x>0;\alpha\:,\:\:\lambda\:>0.$$

Once $\:X=\text{max}({U}_{1},{U}_{3})$ and $\:Y=\text{max}({U}_{2},{U}_{3})$ are defined, the bivariate vector ($\:X$, $\:Y$) is said to have a *BIW* with $\:{\lambda\:}_{1},{\lambda\:}_{2},{\lambda\:}_{3}\:\text{a}\text{n}\text{d}\:\alpha\:$ parameters, as shown by BIW($\:\alpha\:,\:{\lambda\:}_{1},{\lambda\:}_{2},{\lambda\:}_{3})$.

Notably, the shape parameter of the random variables $\:{U}_{1},{U}_{2}\:$ and $\:{U}_{3}$is the same. The distribution of $\:\text{max}(X,Y)$ is also $\:IW(\alpha\:,\:{\lambda\:}_{1}+{\lambda\:}_{2}+{\lambda\:}_{3}$), and this guarantees be marginal distributions for *X* and *Y* are IW $\:IW(\alpha\:,\:{\lambda\:}_{1}+{\lambda\:}_{3})$ and $\:IW(\alpha\:,\:{\lambda\:}_{2}+{\lambda\:}_{3}$). Additionally, $\:{\lambda\:}_{3}$ can be regarded as a control parameter for correlation since it will make the two random variables, *X* and *Y*, dependent when it equals 0. Next, we have the joint cdf of (*X*, *Y*) as described below.

$$\:{F}_{BIW}\left(x,y\right)={F}_{IW}\left(x;{\lambda\:}_{1},\alpha\:\right){F}_{IW}\left(y;{\lambda\:}_{2},\alpha\:\right){F}_{IW}\left(z;{\lambda\:}_{3},\alpha\:\right),$$

where $$\:z=\text{m}\text{i}\text{n}(x,y)$$.

Then,

3 $$\:{F}_{BIW}\left(x,y\right)=\left\{\begin{array}{c}{F}_{IW}\left(x;{\lambda\:}_{13},\alpha\:\right){F}_{IW}\left(y;{\lambda\:}_{2},\alpha\:\right),\:\:x<y\:\\\:{F}_{IW}\left(x;{\lambda\:}_{1},\alpha\:\right){F}_{IW}\left(y;{\lambda\:}_{23},\alpha\:\right),\:\:\:\:\:\:\:\:\:\:x>y\\\:{F}_{IW}\left(x;{\lambda\:}_{123},\alpha\:\right),\:\:\:\:\:\:\:\:\:\:\:\:\:\:\:\:\:\:\:\:\:\:\:\:\:\:\:\:\:\:x=y\end{array}\right.$$

where $$\:\:{\lambda\:}_{13}={\lambda\:}_{1}+{\lambda\:}_{3}$$, $$\:{\lambda\:}_{23}={\lambda\:}_{2}+{\lambda\:}_{3}$$ and $$\:{\lambda\:}_{123}={\lambda\:}_{1}+{\lambda\:}_{2}+{\lambda\:}_{3}$$.

The joint pdf of ($\:X$, $\:Y$) is given as follows

4 $$\:{f}_{BIW}\left(x,y\right)=\left\{\begin{array}{c}{\lambda\:}_{13}\:{\lambda\:}_{2\:}{\alpha\:}^{2}{x}^{-\alpha\:-1}{y}^{-\alpha\:-1}{e}^{-{\lambda\:}_{13}{x}^{-\alpha\:}-{\lambda\:}_{2\:}{y}^{-\alpha\:}},\:\:\:\:\:\:\:\:\:\:\:\:\:\:\:\:\:\:\:\:\:\:\:x<y\:\\\:{\lambda\:}_{23}\:{\lambda\:}_{1\:}{\alpha\:}^{2}{x}^{-\alpha\:-1}{y}^{-\alpha\:-1}{e}^{-{\lambda\:}_{23}{x}^{-\alpha\:}-{\lambda\:}_{1\:}{y}^{-\alpha\:}},\:\:\:\:\:\:\:\:\:\:\:\:\:\:\:\:\:\:\:\:\:\:\:\:x>y\\\:{\lambda\:}_{3}\alpha\:{x}^{-\alpha\:-1}{e}^{-{\lambda\:}_{123}{\:x}^{-\alpha\:}},\:\:\:\:\:\:\:\:\:\:\:\:\:\:\:\:\:\:\:\:\:\:\:\:\:\:\:\:\:\:\:\:\:\:\:\:\:\:\:\:\:\:\:\:\:\:\:\:\:\:\:\:\:\:x=y.\end{array}\right.$$

**Fig. 1:**
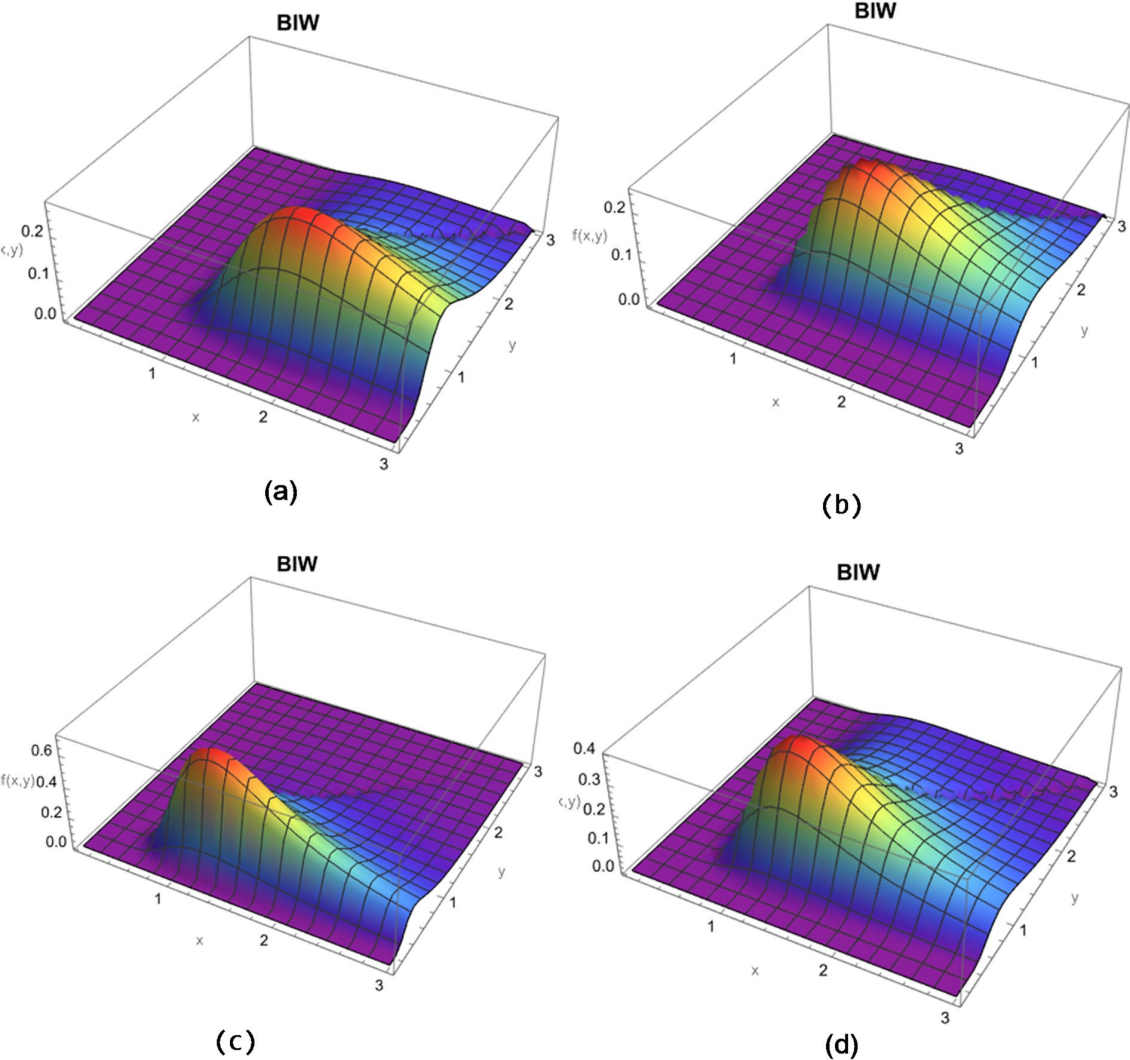
3D plots for the joint pdf of the BIW model based on different parameters values.

Figure 1 shows different graphs for BIW model for specified values for the parameters as (a)$\:{\:\:\:\:\lambda\:}_{1}=5,{\lambda\:}_{2}=0.9,{\lambda\:}_{3}=0.3$ and $\:\alpha\:=2$, (b) $\:{\lambda\:}_{1}=3,{\lambda\:}_{2}=0.5,{\lambda\:}_{3}=2$ ad $\:\alpha\:=2$; (c) $\:{\:\:\:\lambda\:}_{1}=2,{\lambda\:}_{2}=2.2,{\lambda\:}_{3}=0.1$ and $\:\alpha\:=2$ and (d) $\:{\lambda\:}_{1}=2.2,{\lambda\:}_{2}=1,{\lambda\:}_{3}=0.3$ and $\:\alpha\:=2$.

### Data description and likelihood function

In 1980, Reference^7^ made the assumption that while variable X is hard to measure or rank, it is at least easy and affordable to order a sample from the Y population. Thus, a bivariate population can be subjected to the suggested RSS technique in the following manner:

Consider the symbols *m* and *r* such that *m*: is the set size and *r*: is the number of cycles.

Step 1: Select $\:{m}^{2}$ pairs from the population for the $\:{j}^{th}$ cycle.

Step 2: Divide the pairs into the *m* sets at random.

Step 3: Select the $\:{i}^{th}$ order statistics and its concomitant from $\:{i}^{th}$ set, where $\:i=\text{1,2},\dots\:,m$.

Step 4: Repeat steps 1–3 *r* cycle, $\:j=\text{1,2},\dots\:,r$.

Based on RSS scheme, we have the following observations

$$\:\text{F}\text{o}\text{r}\:\text{o}\text{n}\text{e}\:\text{c}\text{y}\text{c}\text{l}\text{e}\:r=1:\:\left[{(x}_{\left(1\right)1},{{y}_{\left[1\right]1}),\:(x}_{\left(2\right)1},{y}_{\left[2\right]1}\right),\dots\:,{(x}_{\left(m\right)1},{y}_{\left[m\right]1}\left)\right]$$

For $$\:r\:$$cycle $$\:r>1$$: $$\:[{(x}_{\left(i\right)j},\:{y}_{\left[i\right]j}),\:i=\text{1,2},\dots\:,m$$, $$\:j=\text{1,2},\dots\:,r$$].

where $\:{X}_{\left(i\right)j}\:$ is the $\:{i}^{th}$ order statistic of *X* in the $\:{j}^{th}$ cycle and $\:{Y}_{\left[i\right]j}$ be its concomitant of $\:Y$.

The corresponding joint pdf of $\:{(x}_{\left(i\right)j},\:{y}_{\left[i\right]j})\:$can be written as

$$\:{f}_{i:m}{(x}_{\left(i\right)j},\:{y}_{\left[i\right]j})=\frac{m!}{(i-1)!(m-i)!}\:{\left[F\right({x}_{\left(i\right)j}\left)\right]}^{i-1}{[1-F({x}_{\left(i\right)j}\left)\right]}^{m-i}\:f{(x}_{\left(i\right)j},\:{y}_{\left[i\right]j})$$

Thus, the likelihood function (LHF) is given as

$$\:L\left({\Theta\:}\right)=\prod\:_{j=1}^{r}\prod\:_{i=1}^{m}{f}_{i:m}{(x}_{\left(i\right)j},\:{y}_{\left[i\right]j}),$$

$$\:L\left({\Theta\:}\right)\propto\:\prod\:_{j=1}^{r}\prod\:_{i=1}^{m}{\left[F\right({(x}_{\left(i\right)j}\left)\right]}^{i-1}{[1-F({(x}_{\left(i\right)j}\left)\right]}^{m-i}\:f({x}_{\left(i\right)j},\:{y}_{\left[i\right]j}).$$

Now, we introduce the LHF in case of Marshall-Olkin bivariate distributions for the first time by taking into consideration the three scenarios of the experiment variables as follows

5 $$\:L\left({\Theta\:}\right)\propto\:\prod\:_{j=1}^{r}\prod\:_{i=1}^{m}{\left[{f}_{1}\right({x}_{\left(i\right)j},\:{y}_{\left[i\right]j}\left)\right]}^{{\delta\:}_{1i\:}}{\left[{f}_{2}\right({x}_{\left(i\right)j},\:{y}_{\left[i\right]j}\left)\right]}^{{\delta\:}_{2i}}{\:\left[{f}_{3}\right({x}_{\left(i\right)j}\left)\right]}^{{\delta\:}_{3i}}{\left[F\right({(x}_{\left(i\right)j}\left)\right]}^{i-1}{\left[\stackrel{-}{F}\right({x}_{\left(i\right)j}\left)\right]}^{m-i}$$

where $\:{\delta\:}_{1i\:},\:{\delta\:}_{2i\:}\:and\:{\delta\:}_{3i\:}$ are event indicators for the $\:{j}^{th}$cycle such that

$$\:{\delta\:}_{1i}\:=\left\{\begin{array}{c}1,\:\:\:\:\:\:\:\:\:\:\:\:\:\:\:\:\:\:{x}_{\left(i\right)j}<{y}_{\left[i\right]j}\\\:0,\:\:\:\:\:\:\:\:\:\:\:\:\:\:\:\:\:\:\:\:\:otherwise\end{array}\right.,\:{\delta\:}_{2i}\:=\left\{\begin{array}{c}1,\:\:\:\:\:\:\:\:\:\:\:\:\:\:\:\:\:\:{x}_{\left(i\right)j}>{y}_{\left[i\right]j}\\\:0,\:\:\:\:\:\:\:\:\:\:\:\:\:\:\:\:\:\:\:\:\:otherwise\end{array}\right.\:\text{a}\text{n}\text{d}\:{\delta\:}_{3i}\:=\left\{\begin{array}{c}1,\:\:\:\:\:\:\:\:\:\:\:\:\:\:\:\:\:\:{x}_{\left(i\right)j}={y}_{\left[i\right]j}\\\:0,\:\:\:\:\:\:\:\:\:\:\:\:\:\:\:\:\:\:\:\:\:otherwise\end{array}\right..$$

So, we can have.

$\:{m}_{1}=\sum\:_{i=1}^{m}{\delta\:}_{1i}$, $\:{m}_{2}=\sum\:_{i=1}^{m}{\delta\:}_{2i}\:$and $\:{m}_{3}=\sum\:_{i=1}^{m}{\delta\:}_{3i}$ such that $\:m={m}_{1}+{m}_{2}+{m}_{3}$.

The choice of set size m and number of cycles r is crucial in RSS designs to balance estimation efficiency and sampling cost. In this study, m is selected as 5, 10, or 15 to rep-resent small to moderate set sizes, as larger m increases ranking accuracy but may introduce judgment errors in practice. The number of cycles r is deter-mined such that the total sample size *n = m × r* aligns with the simulation requirements Recent research highlights the importance of these choices: for instance, Ref^26^. proposed new one- and two-stage RSS variations that optimize m for higher efficiency in mean estimation, while Ref^27^. emphasized adaptive r in power function distributions to reduce variance. These developments underscore how proper selection of m and r enhances precision in bivariate models like BIW, particularly in resource-limited settings.

## Maximum likelihood estimation for BIW model

This section deals with the parametric estimation for the BIW model based on SRS and RSS.

### MLE based on ranked set sampling

In this sub-section, we discussed the MLE for BIW distribution parameters based on RSS. Assume $\:[{(x}_{\left(i\right)j},\:{y}_{\left[i\right]j}),\:i=\text{1,2},\dots\:,m$, $\:j=\text{1,2},\dots\:,r$] denote RSS from the cdf and pdf of the $\:BIW({\lambda\:}_{1},{\lambda\:}_{2},{\lambda\:}_{3},\alpha\:)$ distribution are provided in (3) and (4) are given in (3) and (4), for simplicity assume $\:{{x}_{ij}=x}_{\left(i\right)j}$ and $\:{{y}_{ij}=y}_{\left[i\right]j}$.

The LHF for the sample of size $\:n=rm$

$$\:L\left({\Theta\:}\right)\propto\:\prod\:_{j=1}^{r}\prod\:_{i=1}^{m}{\left[{f}_{1}\right({x}_{ij},\:{y}_{ij}\left)\right]}^{{\delta\:}_{1i\:}}{\left[{f}_{2}\right({x}_{ij},\:{y}_{ij}\left)\right]}^{{\delta\:}_{2i}}{\:\left[{f}_{3}\right({x}_{ij}\left)\right]}^{{\delta\:}_{3i}}{\left[F\right({(x}_{ij}\left)\right]}^{i-1}{\left[\stackrel{-}{F}\right({x}_{ij}\left)\right]}^{m-i}$$

The log-LHF $\:l\left({\Theta\:}\right)=log\:L\left({\Theta\:}\right)$ of the RSS of size $\:n=r\:m$ from BIW distribution is given by:

6 $$\begin{aligned} & l\left({\Theta\:}\right)=rmlog\alpha\:+r{m}_{1}log{\lambda\:}_{13}+r{m}_{1}log{\lambda\:}_{2}+r{m}_{2}log{\lambda\:}_{23}+r{m}_{2}log{\lambda\:}_{1}+r{m}_{3}log{\lambda\:}_{3}\nonumber\\&\quad -(\alpha\:+1)\left\{\sum\:_{j=1}^{r}\sum\:_{i=1}^{m}({\delta\:}_{1i\:}+{\delta\:}_{2i\:}+{\delta\:}_{3i\:})\:log{x}_{ij}\:+\right({\delta\:}_{1i\:}+{\delta\:}_{2i\:}\left)\:log{y}_{ij}\right\}\nonumber\\&\quad -{\lambda\:}_{2}\sum\:_{j=1}^{r}\sum\:_{i=1}^{m}{\delta\:}_{1i\:\:}\:{{y}_{ij}}^{-\alpha\:}\:-{\lambda\:}_{1}\sum\:_{j=1}^{r}\sum\:_{i=1}^{m}{\delta\:}_{2i\:\:}\:{{x}_{ij}}^{-\alpha\:}\:-{\lambda\:}_{23}\sum\:_{j=1}^{r}\sum\:_{i=1}^{m}{\delta\:}_{2i\:\:}\:{{y}_{ij}}^{-\alpha\:}\nonumber\\&\quad -{\lambda\:}_{13}\sum\:_{j=1}^{r}\sum\:_{i=1}^{m}{(\delta\:}_{1i\:\:}+i-1)\:{{x}_{ij}}^{-\alpha\:}-{\lambda\:}_{123}\sum\:_{j=1}^{r}\sum\:_{i=1}^{m}\:{{{\delta\:}_{3i\:}x}_{ij}}^{-\alpha\:}\nonumber\\&\quad \:+\sum\:_{j=1}^{r}\sum\:_{i=1}^{m}\left(m-i\right)log\:\left[1-{e}^{-{\lambda\:}_{13}{{x}_{ij}}^{-\alpha\:}}\right].\end{aligned}$$

The log-LHF’s first derivatives with regard to $\:{\lambda\:}_{1},{\lambda\:}_{2}{,\lambda\:}_{3}$ and $\:\alpha\:$ are as follows:

$$\:\frac{\partial\:l}{\partial\:{\lambda\:}_{1}}=\frac{{r\:m}_{1}}{{\lambda\:}_{13}}+\frac{r\:{m}_{2}}{{\lambda\:}_{1}}-{A}_{1}\left({x}_{ij};{\delta\:}_{1i\:}+{\delta\:}_{2i\:}+{\delta\:}_{3i\:};\alpha\:\right)+{B}_{1}\left({\lambda\:}_{13},\alpha\:\right),$$

$$\:\frac{\partial\:l}{\partial\:{\lambda\:}_{2}}=\frac{{r\:m}_{2}}{{\lambda\:}_{23}}+\frac{r\:{m}_{1}}{{\lambda\:}_{2}}-{A}_{2}\left({x}_{ij};{\delta\:}_{3i\:},\alpha\:\right)-{A}_{2}\left({y}_{ij};{\delta\:}_{1i}+{\delta\:}_{2i\:},\alpha\:\right),$$

$$\:\frac{\partial\:l}{\partial\:{\lambda\:}_{3}}=\frac{{r\:m}_{1}}{{\lambda\:}_{13}}+\frac{{r\:m}_{2}}{{\lambda\:}_{23}}+\frac{r\:{m}_{3}}{{\lambda\:}_{3}}-{A}_{2}\left({y}_{ij};{\delta\:}_{2i\:},\alpha\:\right)-{A}_{1}\left({x}_{ij};{\delta\:}_{1i\:}+{\delta\:}_{3i\:},\alpha\:\right)+{B}_{1}\left({\lambda\:}_{13},\alpha\:\right),$$

$$\begin{aligned}\frac{\partial\:l}{\partial\:\alpha\:}&=\frac{5rm}{\alpha\:}-a\left({x}_{ij};{\delta\:}_{1i\:}+{\delta\:}_{2i\:}+{\delta\:}_{3i\:}\right)+a\left({y}_{ij};{\delta\:}_{1i\:}+{\delta\:}_{2i\:}\right)\nonumber\\ &\quad +{\lambda\:}_{2}{a}_{1}\left({y}_{ij};{\delta\:}_{1i},\alpha\:\right)+{\lambda\:}_{1}{a}_{1}\left({x}_{ij};{\delta\:}_{2i},\alpha\:\right)\nonumber\\ &\quad +{\lambda\:}_{23}{a}_{1}\left({y}_{ij};{\delta\:}_{2i},\alpha\:\right)\:+{\lambda\:}_{123}{a}_{1}\left({x}_{ij};{\delta\:}_{3i},\alpha\:\right)\nonumber\\ &\quad +{\lambda\:}_{13}{a}_{2}\left({x}_{ij};{\delta\:}_{1i},\alpha\:\right)-{B}_{2}\left({\lambda\:}_{13},\alpha\:\right)\end{aligned}$$

where

$$\:{A}_{1}\left({x}_{ij};{\delta\:}_{1i\:},\alpha\:\right)=\sum\:_{j=1}^{r}\sum\:_{i=1}^{m}({\delta\:}_{1i\:}+i-1)\:\:{{x}_{ij}}^{-\alpha\:},$$

$$\:{A}_{2}\left({x}_{ij};{\delta\:}_{1i\:},\alpha\:\right)=\sum\:_{j=1}^{r}\sum\:_{i=1}^{m}\:{{{\delta\:}_{3i\:}x}_{ij}}^{-\alpha\:},$$

$$\:a\left({x}_{ij};{\delta\:}_{1i\:}\right)=\sum\:_{j=1}^{r}\sum\:_{i=1}^{m}{\delta\:}_{1i\:}\:\:log\:{x}_{ij},$$

$$\:{a}_{1}\left({x}_{ij};{\delta\:}_{2i},\alpha\:\right)=\sum\:_{j=1}^{r}\sum\:_{i=1}^{m}{\delta\:}_{2i\:}\:\:{{x}_{ij}}^{-\alpha\:}\:log\:{x}_{ij}\:,$$

$$\:{a}_{2}\left({x}_{ij};{\delta\:}_{1i},\alpha\:\right)=\sum\:_{j=1}^{r}\sum\:_{i=1}^{m}{(\delta\:}_{1i\:}+i-1)\:\:{{x}_{ij}}^{-\alpha\:}\:log\:{x}_{ij}\:,$$

$$\:{B}_{1}\left({\lambda\:}_{13},\alpha\:\right)=\sum\:_{j=1}^{r}\sum\:_{i=1}^{m}(m-i)\frac{{{x}_{ij}}^{-\alpha\:}{e}^{-{\lambda\:}_{13}{{x}_{ij}}^{-\alpha\:}}}{1-{e}^{-{\lambda\:}_{13}{{x}_{ij}}^{-\alpha\:}}},$$

$$\:{B}_{2}\left({\lambda\:}_{13},\alpha\:\right)=\sum\:_{j=1}^{r}\sum\:_{i=1}^{m}(m-i)\frac{{\lambda\:}_{13}{{x}_{ij}}^{-\alpha\:}{e}^{-{\lambda\:}_{13}{{x}_{ij}}^{-\alpha\:}}\:log\:{x}_{ij}}{1-{e}^{-{\lambda\:}_{13}{{x}_{ij}}^{-\alpha\:}}}.$$.

Since the aforementioned set of equations lack an explicit form after being equated to zero, their solutions are determined numerically using the Newton-Raphson method, as will be observed in Sect. 5. $\:{\widehat{\lambda\:}}_{1},{\widehat{\lambda\:}}_{2},{\widehat{\lambda\:}}_{3}$ and $\:\widehat{\alpha\:}$, and α ^ are obtained by solving them all at once.

Using the asymptotic normal distribution of the MLEs, confidence intervals are established for the unknown parameters. The asymptotic variance–covariance matrix of the parameters’ MLEs can be roughly represented by inverting the Fisher information matrix F, which is made up of the negative derivatives of the LHF’s natural logarithm assessed at $\:{(\widehat{\alpha\:},\:\widehat{\lambda\:}}_{1},{{\widehat{\lambda\:}}_{2,}\widehat{\lambda\:}}_{3}$) the parameters’ MLEs.

From (6)’s log-LHF, we now get

$$\:{I}_{11}=\frac{{\partial\:}^{2}l}{\partial\:{\lambda\:}_{1}^{2}}=\frac{-r{m}_{2}}{{\lambda\:}_{1}^{2}}-\frac{{m}_{1}}{{\left({\lambda\:}_{1}+{\lambda\:}_{3}\right)}^{2}}-\phi\:({\lambda\:}_{1},{\lambda\:}_{3},\alpha\:),$$

$$\:{I}_{22}=\frac{{\partial\:}^{2}l}{\partial\:{\lambda\:}_{2}^{2}}=\frac{-{rm}_{1}}{{\lambda\:}_{2}^{2}}-\frac{r{m}_{2}}{{\left({\lambda\:}_{2}+{\lambda\:}_{3}\right)}^{2}},$$

$$\:{I}_{33}=\frac{{\partial\:}^{2}l}{\partial\:{\lambda\:}_{3}^{2}}=-\frac{{rm}_{1}}{{\left({\lambda\:}_{1}+{\lambda\:}_{3}\right)}^{2}}-\frac{r{m}_{2}}{{\left({\lambda\:}_{2}+{\lambda\:}_{3}\right)}^{2}}-\frac{{rm}_{3}}{{\lambda\:}_{3}^{2}}-\phi\:({\lambda\:}_{1},{\lambda\:}_{3},\alpha\:),$$

$$\:{I}_{12}=\frac{{\partial\:}^{2}l}{\partial\:{\lambda\:}_{1}\partial\:{\lambda\:}_{2}}=0,\:{I}_{13}=\frac{{\partial\:}^{2}l}{\partial\:{\lambda\:}_{1}\partial\:{\lambda\:}_{3}}=-\frac{rm}{{\left({\lambda\:}_{1}+{\lambda\:}_{3}\right)}^{2}},\:{I}_{23}=\frac{{\partial\:}^{2}l}{\partial\:{\lambda\:}_{2}\partial\:{\lambda\:}_{3}}=-\frac{r{m}_{2}}{{\left({\lambda\:}_{2}+{\lambda\:}_{3}\right)}^{2}},$$

$$\:{I}_{14}=\frac{{\partial\:}^{2}l}{\partial\:{\lambda\:}_{1}\partial\:\alpha\:}={a}_{2}\left({x}_{ij};{\delta\:}_{1i}+{\delta\:}_{2i}+{\delta\:}_{3i},\alpha\:\right)+\omega\:\left({\lambda\:}_{1},{\lambda\:}_{3},\alpha\:\right),$$

$$\:{I}_{24}=\frac{{\partial\:}^{2}l}{\partial\:{\lambda\:}_{2}\partial\:\alpha\:}={a}_{1}\left({y}_{ij};{\delta\:}_{1i},\alpha\:\right)+{a}_{1}\left({y}_{ij};{\delta\:}_{2i},\alpha\:\right)+{a}_{1}\left({x}_{ij};{\delta\:}_{3i},\alpha\:\right),$$

$$\:{I}_{34}=\frac{{\partial\:}^{2}l}{\partial\:{\lambda\:}_{3}\partial\:\alpha\:}={a}_{1}\left({y}_{ij};{\delta\:}_{2i},\alpha\:\right)+{a}_{2}\left({x}_{ij};{\delta\:}_{1i}+{\delta\:}_{3i},\alpha\:\right)+\omega\:\left({\lambda\:}_{1},{\lambda\:}_{3},\alpha\:\right),$$

$$\begin{aligned}{I}_{44}&=\frac{{\partial\:}^{2}l}{\partial\:{\alpha\:}^{2}}=-\frac{5rm}{{\alpha\:}^{2}}-{\lambda\:}_{2}{a}_{3}\left({y}_{ij};{\delta\:}_{1i},\alpha\:\right)-{\lambda\:}_{1}{a}_{3}\left({x}_{ij};{\delta\:}_{2i},\alpha\:\right)\nonumber\\&\quad -{\lambda\:}_{23}{a}_{3}\left({y}_{ij};{\delta\:}_{2i},\alpha\:\right)-{\lambda\:}_{123}{a}_{3}\left({x}_{ij};{\delta\:}_{3i},\alpha\:\right)\nonumber\\&\quad -{\lambda\:}_{123}{a}_{4}\left({x}_{ij};{\delta\:}_{1i},\alpha\:\right)-\:\omega\:\left({\lambda\:}_{1},{\lambda\:}_{3},\alpha\:\right)\end{aligned}$$

where

$$\:\phi\:\left({\lambda\:}_{1},{\lambda\:}_{3},\alpha\:\right)=\sum\:_{j=1}^{r}\sum\:_{i=1}^{m}(m-i)\frac{{x}_{ij}^{-\alpha\:}{e}^{-({\lambda\:}_{1}+{\lambda\:}_{3}){x}_{ij}^{-\alpha\:}}}{1-{e}^{-({\lambda\:}_{1}+{\lambda\:}_{3}){x}_{ij}^{-\alpha\:}}},$$

$$\:{a}_{1}\left({x}_{ij};{\delta\:}_{2i},\alpha\:\right)=\sum\:_{j=1}^{r}\sum\:_{i=1}^{m}{\delta\:}_{2i\:}\:\:{{x}_{ij}}^{-\alpha\:}\:log\:{x}_{ij}\:,$$

$$\:{a}_{2}\left({x}_{ij};{\delta\:}_{1i},\alpha\:\right)=\sum\:_{j=1}^{r}\sum\:_{i=1}^{m}{(\delta\:}_{1i\:}+i-1)\:\:{{x}_{ij}}^{-\alpha\:}\:log\:{x}_{ij}\:$$

$$\:{a}_{3}\left({x}_{ij};{\delta\:}_{1i},\alpha\:\right)=\sum\:_{j=1}^{r}\sum\:_{i=1}^{m}{\delta\:}_{1i}{x}_{ij}^{-\alpha\:}\:{\left(log{x}_{ij}\right)}^{2},$$

$$\:{a}_{4}\left({x}_{ij};{\delta\:}_{1i},\alpha\:\right)=\sum\:_{j=1}^{r}\sum\:_{i=1}^{m}{(\delta\:}_{1i}+i-1){x}_{ij}^{-\alpha\:}\:{\left(log{x}_{ij}\right)}^{2},$$

$$\:\omega\:\left({\lambda\:}_{1},{\lambda\:}_{3},\alpha\:\right)={\lambda\:}_{13}\sum\:_{j=1}^{r}\sum\:_{i=1}^{m}(m-i)\frac{{x}_{ij}^{-\alpha\:}{\left(log{x}_{ij}\right)}^{2}{e}^{-{\lambda\:}_{13}{x}_{ij}^{-\alpha\:}}\left\{\right(1-{e}^{-{\lambda\:}_{13}{x}_{ij}^{-\alpha\:}}\left)\right({x}_{ij}^{-\alpha\:}-1)+{x}_{ij}^{-\alpha\:}{e}^{-{\lambda\:}_{13}{x}_{ij}^{-\alpha\:}}\}}{{(1-{e}^{-{\lambda\:}_{13}{x}_{ij}^{-\alpha\:}})}^{2}}.$$

The asymptotic variance-covariance matrix can therefore be expressed as follows.

$$\:{F}^{-1}={{\left(\begin{array}{c}-{I}_{11}\\\:-{I}_{21}\\\:-{I}_{31}\\\:-{I}_{41}\end{array}\begin{array}{c}-{I}_{12}\\\:-{I}_{22}\\\:-{I}_{32}\\\:-{I}_{42}\end{array}\begin{array}{c}-{I}_{13}\\\:-{I}_{23}\\\:-{I}_{33}\\\:-{I}_{43}\end{array}\begin{array}{c}-{I}_{14}\\\:-{I}_{24}\\\:-{I}_{34}\\\:-{I}_{44}\end{array}\right)}^{-1}}_{\left|({\widehat{\lambda\:}}_{1},{\widehat{\lambda\:}}_{2},{\widehat{\lambda\:}}_{3},\widehat{\alpha\:})\right.}$$

For $\:{\lambda\:}_{1},{\lambda\:}_{2}{,\lambda\:}_{3}$ and $\:\alpha\:$, the estimated confidence intervals are:

7 $$\:{\widehat{\lambda\:}}_{1}\pm\:{z}_{\frac{\gamma\:}{2}}\sqrt{{v}_{11}},{\widehat{\lambda\:}}_{2}\pm\:{z}_{\frac{\gamma\:}{2}}\sqrt{{v}_{22}},{\widehat{\lambda\:}}_{3}\pm\:{z}_{\frac{\gamma\:}{2}}\sqrt{{v}_{33}}\:\text{a}\text{n}\text{d}\:\widehat{\alpha\:}\pm\:{z}_{\frac{\gamma\:}{2}}\sqrt{{v}_{44}}.$$

where $\:{z}_{\frac{\gamma\:}{2}}$ is the percentile of the standard normal distribution with right tail $\:\frac{\gamma\:}{2}$ and entries on the major diagonal of the variance-covariance matrix are $\:{v}_{11},{v}_{22},{v}_{33}$ and $\:{v}_{44}$.

### MLE based on simple random sample

Let that $\:i=1\dots\:n$ is the experiment, and that there are $\:n$ independent pairings of components ($\:{X}_{i},{Y}_{i}$), each of which has a $\:BIW({\lambda\:}_{1},{\lambda\:}_{2},{\lambda\:}_{3},\alpha\:)$ distribution.

The LHF of the sample of size *n* is given by

$$\:L\left(\theta\:\right)=\:\prod\:_{i=1}^{n}{[{f}_{1}{(x}_{1i},{x}_{2i})]}^{{\delta\:}_{1i}}\:{[{f}_{2}{(x}_{1i},{x}_{2i})]}^{{\delta\:}_{2i}\:}{[{f}_{3}{(x}_{1i},{x}_{2i})]}^{{\delta\:}_{3i}}.$$

where $\:{n}_{1}=\sum\:_{i=1}^{n}{\delta\:}_{1i}$, $\:{n}_{2}=\sum\:_{i=1}^{n}{\delta\:}_{2i}\:$and $\:{n}_{3}=\sum\:_{i=1}^{n}{\delta\:}_{3i}$ such that $\:n={n}_{1}+{n}_{2}+{n}_{3}$.

The log-LHF of the SRS of size *n* from BIW distribution is given by

8 $$\begin{aligned}l\left({\Theta\:}\right)&=({2n}_{1}+2{n}_{2}+{n}_{3})log\alpha\:+{n}_{1}log{\lambda\:}_{13}+{n}_{1}log{\lambda\:}_{2}+{n}_{2}log{\lambda\:}_{23}+{n}_{2}log{\lambda\:}_{1}+{n}_{3}log{\lambda\:}_{3}\nonumber\\ &\quad -(\alpha\:+1)\left\{\sum\:_{i=1}^{n}({\delta\:}_{1i\:}+{\delta\:}_{2i\:}+{\delta\:}_{3i\:})\:log{x}_{i}+{\delta\:}_{1i\:}\:log{y}_{i}\right\}-{\lambda\:}_{13}\sum\:_{i=1}^{n}{\delta\:}_{1i\:\:}\:{{x}_{i}}^{-\alpha\:}\nonumber\\ &\quad -{\lambda\:}_{2}\sum\:_{i=1}^{n}{\delta\:}_{1i\:\:}\:{{y}_{i}}^{-\alpha\:}-{\lambda\:}_{23}\sum\:_{i=1}^{n}{\delta\:}_{2i\:\:}\:{{y}_{i}}^{-\alpha\:}-{\lambda\:}_{1}\sum\:_{i=1}^{n}{\delta\:}_{2i\:\:}\:{{x}_{i}}^{-\alpha\:}-{\lambda\:}_{123}\sum\:_{i=1}^{n}{\delta\:}_{3i\:\:}\:{{x}_{i}}^{-\alpha\:}.\end{aligned}$$

The 1st derivatives of the log- LHF with respect to $\:{\lambda\:}_{1},{\lambda\:}_{2}{,\lambda\:}_{3}$ and $\:\alpha\:$ are as follows

$$\:\frac{\partial\:l}{\partial\:{\lambda\:}_{1}}=\frac{{n}_{1}}{{\lambda\:}_{13}}+\frac{{n}_{2}}{{\lambda\:}_{1}}-\sum\:_{i=1}^{n}{(\delta\:}_{1i\:\:}+{\delta\:}_{2i\:\:}+{\delta\:}_{3i\:\:})\:{{x}_{i}}^{-\alpha\:},$$

$$\:\frac{\partial\:l}{\partial\:{\lambda\:}_{2}}=\frac{{n}_{1}}{{\lambda\:}_{2}}+\frac{{n}_{2}}{{\lambda\:}_{23}}-\sum\:_{i=1}^{n}{\delta\:}_{3i\:\:}\:{{x}_{i}}^{-\alpha\:}-\sum\:_{i=1}^{n}{(\delta\:}_{1i\:\:}+{\delta\:}_{2i\:\:})\:{{y}_{i}}^{-\alpha\:},$$

$$\:\frac{\partial\:l}{\partial\:{\lambda\:}_{3}}=\frac{{n}_{1}}{{\lambda\:}_{13}}+\frac{{n}_{2}}{{\lambda\:}_{23}}+\frac{{n}_{3}}{{\lambda\:}_{3}}-\sum\:_{i=1}^{n}{(\delta\:}_{1i\:\:}+{\delta\:}_{3i\:\:})\:{{x}_{i}}^{-\alpha\:}-\sum\:_{i=1}^{n}{\delta\:}_{2i\:\:}\:{{y}_{i}}^{-\alpha\:},$$

$$\begin{aligned}\:\frac{\partial\:l}{\partial\:\alpha\:}&=\frac{{2n}_{1}+{2n}_{2}+{n}_{3}}{\alpha\:}+{\lambda\:}_{13}\sum\:_{i=1}^{n}{\delta\:}_{1i\:\:}\:{{x}_{i}}^{-\alpha\:}log{x}_{i}\nonumber\\ &\quad +{\lambda\:}_{2}\sum\:_{i=1}^{n}{\delta\:}_{1i\:\:}\:{{y}_{i}}^{-\alpha\:}log{y}_{i}\:+{\lambda\:}_{23}\sum\:_{i=1}^{n}{\delta\:}_{2i\:\:}\:{{y}_{i}}^{-\alpha\:}log{y}_{i}\nonumber\\ &\quad + {\lambda\:}_{1}\sum\:_{i=1}^{n}{\delta\:}_{2i\:\:}\:{{x}_{i}}^{-\alpha\:}log{x}_{i}+{\lambda\:}_{123}\sum\:_{i=1}^{n}{\delta\:}_{3i\:\:}\:{{x}_{i}}^{-\alpha\:}log{x}_{i}\nonumber\\ &\quad -\sum\:_{i=1}^{n}{(\delta\:}_{1i\:\:}+{\delta\:}_{2i\:\:}+{\delta\:}_{3i\:\:})\:log{x}_{i}+\sum\:_{i=1}^{n}{(\delta\:}_{1i\:\:}+{\delta\:}_{2i\:\:})\:log{y}_{i}\end{aligned}$$

As will be seen in Sect. 5, the Newton-Raphson method is used to numerically determine the solutions to the preceding set of equations because, regrettably, it is discovered that they lack an explicit form after being equated to zero once more. To obtain $\:{\widehat{\lambda\:}}_{1},{\widehat{\lambda\:}}_{2},{\widehat{\lambda\:}}_{3}$ and $\:\widehat{\alpha\:}$, they are solved concurrently.

## Bayesian estimation for BIW model

In this section we consider the Bayesian estimation for the BIW distribution parameters based on RSS and SRS. We obtained the Bayes estimators under the squared error loss function for both cases.

### Ranked set sampling

The Bayesian estimators for the BIW distribution are derived explicitly in case of RSS in this sub-section as follows.

**The prior assumption**:

The same conjugate prior on $\:{\lambda\:}_{1},{\lambda\:}_{2}$ and $\:{\lambda\:}_{3}$ is assumed when the shape parameter α is known.

Assume that $\:{\lambda\:}_{1},{\lambda\:}_{2}$ and $\:{\lambda\:}_{3}$ are independent and come in the following gamma distribution.

$$\:{\pi\:}_{i}\left({\lambda\:}_{i}\right)=\frac{{b}^{{a}_{i}}}{{\Gamma\:}\left({a}_{i}\right)}\:{{\lambda\:}_{i}}^{{a}_{i}-1}{e}^{-b{\lambda\:}_{i}}\:,\:i=\text{1,2},3\:,{\lambda\:}_{i}>0\:$$

$$\:{\lambda\:}_{1},{\lambda\:}_{2}$$ and $$\:{\lambda\:}_{3}{\prime\:}s\:c\:\text{j}\text{o}\text{i}\text{n}\text{t}\:\text{p}\text{r}\text{i}\text{o}\text{r}\:\text{d}\text{e}\text{n}\text{s}\text{i}\text{t}\text{y}$$

$$\:{\pi\:}_{0}\left({\lambda\:}_{1},{\lambda\:}_{2},{\lambda\:}_{3}\right)=\prod\:_{i=1}^{3}\frac{{b}^{{a}_{i}}}{{\Gamma\:}\left({a}_{i}\right)}\:{{\lambda\:}_{i}}^{{a}_{i}-1}{e}^{-b\:{\lambda\:}_{i}}$$.

**Bayesian Inference and Posterior Analysis**.

Give us a bivariate RSS from $\:BIW({\lambda\:}_{1},{\lambda\:}_{2},{\lambda\:}_{3},\alpha\:)$ distribution and It’s indicated as follows:

$\:D=[{(x}_{\left(i\right)j},\:{y}_{\left[i\right]j}),\:i=\text{1,2},\dots\:,m$, $\:j=\text{1,2},\dots\:,r$]. Again, for simplicity assume $\:{{x}_{ij}=x}_{\left(i\right)j}$ and $\:{{y}_{ij}=y}_{\left[i\right]j}$.

Let $$\:m={m}_{1}+{m}_{2}+{m}_{3}$$, $$\:{\lambda\:}_{123}={\lambda\:}_{1}+{\lambda\:}_{2}+{\lambda\:}_{3}$$, $$\:{\lambda\:}_{13}={\lambda\:}_{1}+{\lambda\:}_{3}$$and $$\:{\lambda\:}_{23}={\lambda\:}_{2}+{\lambda\:}_{3}.$$.

The LHF in (6) can therefore be rewritten

$$\:L(D\backslash\:{\Theta\:})=\:Exp\left(\text{log}L(D\backslash\:{\Theta\:})\right)$$

$$\:L(D\backslash\:{\Theta\:})=\sum\:_{k=1}^{{rm}_{1}}\sum\:_{s=1}^{{rm}_{2}}\sum\:_{l=1}^{m-i}\left(\begin{array}{c}{rm}_{1}\\\:k\end{array}\right)\left(\begin{array}{c}{rm}_{2}\\\:s\end{array}\right)\left(\begin{array}{c}m-i\\\:l\end{array}\right){(-1)}^{l}{{\lambda\:}_{1}}^{{rm}_{2}+k}{{\lambda\:}_{2}}^{r{m}_{1}+s}{.{\lambda\:}_{3}}^{rm-k-s}$$

9 $$\:Exp\{{-\lambda\:}_{2}{Z}_{1}\left(\alpha\:\right)-{\lambda\:}_{1}{Z}_{2}\left(\alpha\:\right){-\lambda\:}_{23}{Z}_{3}\left(\alpha\:\right)-{\lambda\:}_{13}{Z}_{4}\left(\alpha\:\right){-\lambda\:}_{123}{Z}_{5}\left(\alpha\:\right)-(\alpha\:+1\left){Z}_{6}\right\}$$

where $$\:{Z}_{1}\left(\alpha\:\right)=\sum\:_{j=1}^{r}\sum\:_{i=1}^{m}{\delta\:}_{1i}{y}_{ij}^{-\alpha\:},$$ $$\:{Z}_{2}\left(\alpha\:\right)=\sum\:_{j=1}^{r}\sum\:_{i=1}^{m}{\delta\:}_{2i}{x}_{ij}^{-\alpha\:},$$ $$\:{Z}_{3}\left(\alpha\:\right)=\sum\:_{j=1}^{r}\sum\:_{i=1}^{m}{\delta\:}_{2i}{y}_{ij}^{-\alpha\:},$$ $$\:{Z}_{4}\left(\alpha\:\right)=\sum\:_{j=1}^{r}\sum\:_{i=1}^{m}{(\delta\:}_{1i}+i+l-1){x}_{ij}^{-\alpha\:},$$ $$\:{Z}_{5}\left(\alpha\:\right)=\sum\:_{j=1}^{r}\sum\:_{i=1}^{m}{\delta\:}_{3i}{x}_{ij}^{-\alpha\:}$$ and $$\:{Z}_{6}=\sum\:_{j=1}^{r}\sum\:_{i=1}^{m}\{{(\delta\:}_{1i}+{\delta\:}_{2i}+{\delta\:}_{3i})log{x}_{ij}+{(\delta\:}_{1i}+{\delta\:}_{2i}\left)log{y}_{ij}\right\}.$$

Since Bayes theory states that: $\:f\left(D,{\Theta\:}\right)=\:L(D\backslash\:{\Theta\:})\:{\pi\:}_{0}\left({\Theta\:}\right)\:$ and since $\:f\left(D\right)=\int\:f\left(D\backslash\:{\Theta\:}\right)d{\Theta\:}=\int\:{\pi\:}_{0}\left({\Theta\:}\right)L(D\backslash\:{\Theta\:})d{\Theta\:}$.

Therefore, given the data D, $\:{\Theta\:}=(\alpha\:,\:{\lambda\:}_{1},{\lambda\:}_{2},{\lambda\:}_{3})$ is joint posterior density function, represented by $\:{\pi\:}_{1}(\:{\Theta\:}\backslash\:D)$, may be expressed as

$$\:{\pi\:}_{1}(\:{\Theta\:}\backslash\:D)\:=\frac{f\left(D,{\Theta\:}\right)}{f\left(D\right)},$$

$$\:{\pi\:}_{1}\left(\:{\Theta\:}\backslash\:D\right)\propto\:\sum\:_{k=1}^{{rm}_{1}}\sum\:_{s=1}^{{rm}_{2}}\sum\:_{l=1}^{m-i}{w}_{ksl\:\:}Gamma\left[{\lambda\:}_{1};{a}_{1k},{b}_{1}+{T}_{1}\left(\alpha\:\right)\right].$$

10 $$\:Gamma\:[{\lambda\:}_{2};\:{a}_{2s\:},\:{b}_{2}+{T}_{2}(\alpha\:\left)\right].\:Gamma\:[{\lambda\:}_{3}\:;\:{a}_{3s},\:{b}_{3}+{T}_{3}(\alpha\:\left)\right],$$

where $\:{w}_{ksl\:\:}=\frac{{C}_{ksl\:\:}}{\sum\:_{k=1}^{{rm}_{1}}\sum\:_{s=1}^{{rm}_{2}}\sum\:_{l=1}^{m-i}{C}_{ksl\:\:}},$ and $$\:{C}_{ksl}={(-1)}^{l}\left(\begin{array}{c}{rm}_{1}\\\:k\end{array}\right)\left(\begin{array}{c}{rm}_{2}\\\:s\end{array}\right)\left(\begin{array}{c}m-i\\\:l\end{array}\right).\frac{{\Gamma\:}\left({a}_{1k}\right)}{{[{b}_{1}+{T}_{1}(\alpha\:\left)\right]}^{{a}_{1k}}}.$$ $\:\:\frac{{\Gamma\:}{a}_{2s\:})}{{[{b}_{2}+{T}_{2}(\alpha\:\left)\right]}^{{a}_{2s\:}}}.$ $\:\:\frac{{\Gamma\:}\left({a}_{3s}\right)}{{[{b}_{3}+{T}_{3}(\alpha\:\left)\right]}^{{a}_{3s}}},$ $\:{T}_{1}\left(\alpha\:\right)={Z}_{2}\left(\alpha\:\right)+{Z}_{4}\left(\alpha\:\right)+{Z}_{5}\left(\alpha\:\right),$ $\:{T}_{2}\left(\alpha\:\right)={Z}_{1}\left(\alpha\:\right)+{Z}_{3}\left(\alpha\:\right)+{Z}_{5}\left(\alpha\:\right),$ $\:{T}_{3}\left(\alpha\:\right)={Z}_{3}\left(\alpha\:\right)+{Z}_{4}\left(\alpha\:\right)+{Z}_{5}\left(\alpha\:\right),$ $\:{a}_{1k}={a}_{1}+r{m}_{2}+k,$ $\:{a}_{2s\:}={a}_{2}+{rm}_{1}+s,$ and $\:{{a}_{3s}=a}_{3}+{rm}_{1}+{rm}_{2}-s.$

Therefore, assuming that $\:{\lambda\:}_{1},{\lambda\:}_{2}$ and $\:{\lambda\:}_{3}$ are independent, α is assumed to be known. The Bayes estimators of $\:{\lambda\:}_{1},{\lambda\:}_{2}$ and $\:{\lambda\:}_{3}$ may be explicitly calculated under the square error loss function (SELF) using (10) and will look like this.

$${\check{\lambda}}_{1}=\frac{1}{{b}_{1}+{T}_{1}}\sum\:_{k=1}^{{rm}_{1}}\sum\:_{s=1}^{{rm}_{2}}\sum\:_{l=1}^{m-i}{w}_{ksl\:\:}{a}_{1k},$$

$${\check{\lambda}}_{2}=\frac{1}{{b}_{2}+{T}_{2}}\sum\:_{k=1}^{{rm}_{1}}\sum\:_{s=1}^{{rm}_{2}}\sum\:_{l=1}^{m-i}{w}_{ksl\:\:}{a}_{2s},$$

and

$${\check{\lambda}}_{3}=\frac{1}{{b}_{3}+{T}_{3}}\sum\:_{k=1}^{{rm}_{1}}\sum\:_{s=1}^{{rm}_{2}}\sum\:_{l=1}^{m-i}{w}_{ksl\:\:}{a}_{3s}.$$

Using the Markov Chain Monte Carlo (MCMC) technique, we can generate samples from the posterior distributions and then compute the Bayes estimators of the individual parameters. Using the SELF, we can thus derive the Bayes estimates for $\:{\Theta\:}=(\alpha\:,\:{\lambda\:}_{1},{\lambda\:}_{2},{\lambda\:}_{3})$ the vector of unknown parameters, assuming that α is unknown.

### Simple random samples

In this sub-section the Bayesian estimators are derived explicitly for the BIW distribution under the SELF in case of SRS. We consider the same prior assumption as in the RSS case with known shape parameter $\:\alpha\:$.

So, the joint prior density of the independent parameters $\:{\lambda\:}_{1},{\lambda\:}_{2}$ and $\:{\lambda\:}_{3},$ is given as

$$\:{\pi\:}_{0}\left({\lambda\:}_{1},{\lambda\:}_{2},{\lambda\:}_{3}\right)=\prod\:_{i=1}^{3}\frac{{b}^{{a}_{i}}}{{\Gamma\:}\left({a}_{i}\right)}\:{{\lambda\:}_{i}}^{{a}_{i}-1}{e}^{-b\:{\lambda\:}_{i}}.$$

Suppose our bivariate SRS from $\:BIW(\alpha\:,\:{\lambda\:}_{1},{\lambda\:}_{2},{\lambda\:}_{3})$ distribution and it is denoted as

$$\:D=[{(x}_{i},\:{y}_{i}),\:i=\text{1,2},\dots\:,n].$$

Let $$\:n={n}_{1}+{n}_{2}+{n}_{3}$$, $$\:{\lambda\:}_{123}={\lambda\:}_{1}+{\lambda\:}_{2}+{\lambda\:}_{3}$$, $$\:{\lambda\:}_{13}={\lambda\:}_{1}+{\lambda\:}_{3}$$, $$\:{\lambda\:}_{23}={\lambda\:}_{2}+{\lambda\:}_{3}$$

and $$\:{\Theta\:}=\left({\lambda\:}_{1},{\lambda\:}_{2},{\lambda\:}_{3}\right)$$.

LHF in (8) can be rewritten as

$$\:L(D\backslash\:{\Theta\:})=\:Exp\left(\text{log}L(D\backslash\:{\Theta\:})\right)$$

$$\:L(D\backslash\:{\Theta\:})=\sum\:_{s=1}^{{n}_{2}}\sum\:_{k=1}^{{n}_{1}}\left(\begin{array}{c}{n}_{1}\\\:k\end{array}\right)\left(\begin{array}{c}{n}_{2}\\\:s\end{array}\right){{\lambda\:}_{1}}^{{n}_{2}+k}{{\lambda\:}_{2}}^{{n}_{1}+s}{.{\lambda\:}_{3}}^{n-k-s}$$

11 $$\:Exp\{{-\lambda\:}_{2}{Z}_{1}\left(\alpha\:\right)-{\lambda\:}_{1}{Z}_{2}\left(\alpha\:\right){-\lambda\:}_{23}{Z}_{3}\left(\alpha\:\right)-{\lambda\:}_{13}{Z}_{4}\left(\alpha\:\right){-\lambda\:}_{123}{Z}_{5}\left(\alpha\:\right)-(\alpha\:+1\left){Z}_{6}\right\}$$

where $$\:{Z}_{1}\left(\alpha\:\right)=\sum\:_{i=1}^{n}{\delta\:}_{1i}{y}_{i}^{-\alpha\:},$$ $$\:{Z}_{2}\left(\alpha\:\right)=\sum\:_{i=1}^{n}{\delta\:}_{2i}{x}_{i}^{-\alpha\:},$$ $$\:{Z}_{3}\left(\alpha\:\right)=\sum\:_{i=1}^{n}{\delta\:}_{2i}{y}_{i}^{-\alpha\:},$$ $$\:{Z}_{4}\left(\alpha\:\right)=\sum\:_{i=1}^{n}{\delta\:}_{1i}{x}_{i}^{-\alpha\:},$$ $$\:{Z}_{5}\left(\alpha\:\right)=\sum\:_{i=1}^{n}{\delta\:}_{3i}{x}_{i}^{-\alpha\:}$$ and $$\:{Z}_{6}=\sum\:_{i=1}^{n}\{{(\delta\:}_{1i}+{\delta\:}_{2i}+{\delta\:}_{3i})log{x}_{i}+{(\delta\:}_{1i}+{\delta\:}_{2i}\left)log{y}_{i}\right\}.$$

Furthermore, the joint posterior density function $\:{\pi\:}_{1}(\:{\Theta\:}\backslash\:D)$ of $\:{\Theta\:}=({\lambda\:}_{1},{\lambda\:}_{2},{\lambda\:}_{3})$ is given by gamma densities as follows

$$\:{\pi\:}_{1}\left(\:{\Theta\:}\backslash\:D\right)\propto\:\sum\:_{s=1}^{{n}_{2}}\sum\:_{k=1}^{{n}_{1}}{Q}_{ks\:\:}Gamma\left[{\lambda\:}_{1};{a}_{1k},\:\:{b}_{1}+{T}_{1}\left(\alpha\:\right)\right].$$

12 $$\:Gamma\:\left[{\lambda\:}_{2};\:{a}_{2s\:},\:\:{b}_{2}+{T}_{2}\left(\alpha\:\right)\right].\:Gamma\:\left[{\lambda\:}_{3}\:;\:{a}_{3ks},\:\:{b}_{3}+{T}_{3}\left(\alpha\:\right)\right],$$

where $$\:{Q}_{ks\:\:}=\frac{{q}_{ks\:\:}}{\sum\:_{s=1}^{{n}_{2}}\sum\:_{k=1}^{{n}_{1}}{q}_{ks\:\:}}$$, and $$\:{q}_{ks}=\left(\begin{array}{c}{n}_{1}\\\:k\end{array}\right)\left(\begin{array}{c}{n}_{2}\\\:s\end{array}\right).\frac{{\Gamma\:}\left({a}_{1k}\right)}{{[{b}_{1}+{T}_{1}(\alpha\:\left)\right]}^{{a}_{1k}}}$$. $$\:\:\frac{{\Gamma\:}{a}_{2s\:})}{{[{b}_{2}+{T}_{2}(\alpha\:\left)\right]}^{{a}_{2s\:}}}$$. $$\:\:\frac{{\Gamma\:}\left({a}_{3sk}\right)}{{[{b}_{3}+{T}_{3}(\alpha\:\left)\right]}^{{a}_{3sk}}}$$.

$$\:{T}_{1}\left(\alpha\:\right)={Z}_{2}\left(\alpha\:\right)+{Z}_{4}\left(\alpha\:\right)+{Z}_{5}\left(\alpha\:\right),$$

$$\:{T}_{2}\left(\alpha\:\right)={Z}_{1}\left(\alpha\:\right)+{Z}_{3}\left(\alpha\:\right)+{Z}_{5}\left(\alpha\:\right),$$

$$\:{T}_{3}\left(\alpha\:\right)={Z}_{3}\left(\alpha\:\right)+{Z}_{4}\left(\alpha\:\right)+{Z}_{5}\left(\alpha\:\right),$$

$$\:{a}_{1k}={a}_{1}+{n}_{2}+k,$$

$$\:{a}_{2s\:}={a}_{2}+{n}_{1}+s,$$

and

$$\:{{a}_{3sk}=a}_{3}+n-s-k$$

Hence, Using the Bayes estimators for the parameters $\:{\lambda\:}_{1},{\lambda\:}_{2}$and $\:{\lambda\:}_{3}$ under the SELF based on SRS are given as follows:

$${\check{\lambda}}_{1} = \frac{1}{{{b_1} + {T_1}}}\mathop \sum \limits_{s = 1}^{{n_2}} \mathop \sum \limits_{k = 1}^{{n_1}} {Q_{ks~~}}{a_{1k}}$$

$${\check{\lambda}}_{2}=\frac{1}{{b}_{2}+{T}_{2}}\sum\:_{s=1}^{{n}_{2}}\sum\:_{k=1}^{{n}_{1}}{Q}_{ks\:\:}{a}_{2s},$$

and

$${\check{\lambda}}_{3}=\frac{1}{{b}_{3}+{T}_{3}}\sum\:_{s=1}^{{n}_{2}}\sum\:_{k=1}^{{n}_{1}}{Q}_{ks\:\:}{a}_{3sk}.$$

Again, as in RSS if we assume that$\:\:\alpha\:$ is unknown a Markov Chain Monte Carlo (MCMC) technique is used to generate samples from the posterior distributions and then compute the Bayes estimators of the individual parameters.

### Prior sensitivity analysis and justification

This discusses the sensitivity of Bayesian estimates for $\:{\widehat{\lambda\:}}_{1},{\widehat{\lambda\:}}_{2}\:and\:{\widehat{\lambda\:}}_{3}$ to gamma priors with shape parameters $\:a_{1}=2,\:a_{2}=2,\:a_{3}=2$ and scale parameter$\:\:b=1.$ Gamma priors were chosen for their conjugacy with the BIW likelihood, enabling analytical posterior derivations. The additional parameters $\:c=2\:and\:d=1$ were used as shape and scale for an alternative prior specification to compare performance.

**Table 1:** Prior sensitivity analysis for bayesian Estimation (parameter set (0.2, 0.8, 0.4), m = 10).

Prior specification	Parameter	MSE (RSS)	Bias (RSS)	MSE (SRS)	Bias (SRS)
$\:{a}_{1}=2,\:{a}_{2}=2,\:$ $\:a_{3}=2,\:b=1$	$\:{\widehat{\lambda\:}}_{1}$	0.15	0.07	0.35	0.12
	$\:{\widehat{\lambda\:}}_{2}$	0.015	0.01	0.035	0.025
	$\:{\widehat{\lambda\:}}_{3}$	0.005	0.003	0.02	0.015
$\:{a}_{1}=1,\:{a}_{2}=1,\:$ $\:a_{3}=1,\:b=0.5$	$\:{\widehat{\lambda\:}}_{1}$	0.22	0.11	0.42	0.18
	$\:{\widehat{\lambda\:}}_{2}$	0.04	0.03	0.08	0.05
	$\:{\widehat{\lambda\:}}_{3}$	0.025	0.02	0.06	0.035
$\:{a}_{1}=c=2,\:$ $\:{a}_{2}=c=2,\:$ $\:{a}_{3}=c=2,$ $\:\:b=d=1$	$\:{\widehat{\lambda\:}}_{1}$	0.148	0.068	0.345	0.118
	$\:{\widehat{\lambda\:}}_{2}$	0.014	0.009	0.034	0.024
	$\:{\widehat{\lambda\:}}_{3}$	0.004	0.002	0.019	0.014

Table 1 presents the sensitivity analysis results for Bayesian estimation of BIW parameters ( $\:{\widehat{\lambda\:}}_{1}=0.2,\:{\widehat{\lambda\:}}_{2}=0.8\:and\:{\widehat{\lambda\:}}_{3}=0.4$) using three gamma prior specifications: (1) $\:{a}_{1}=2,\:{a}_{2}=2,\:a_{3}=2,\:b=1$ (baseline, moderately informative), (2) $\:{a}_{1}=1,\:{a}_{2}=1,\:a_{3}=1,\:b=0.5$ (non-informative), and (3) $\:{a}_{1}=c=2,\:{a}_{2}=c=2,\:{a}^{3}=c=2,\:b=1$ (alternative, moderately informative). The results confirm the superiority of RSS over SRS, with significantly lower MSE and Bias under RSS across all priors.

Baseline Priors ($\:{a}_{1}=2,\:{a}_{2}=2,\:a_{3}=2,\:b=1$. These priors exhibit excellent performance, with very low MSE (0.150 for $\:{\widehat{\lambda\:}}_{1}$, 0.015 for $\:{\widehat{\lambda\:}}_{2}$, 0.005 for $\:{\widehat{\lambda\:}}_{3}$ under RSS) and minimal Bias (0.070 for λ_1_, 0.010 for λ_2_, 0.003 for λ_0_). Compared to SRS, MSE and Bias are substantially higher (e.g., 0.350 and 0.120 for $\:{\widehat{\lambda\:}}_{1}$), highlighting RSS’s efficiency.

Non-Informative Priors ($\:{a}_{1}=1,\:{a}_{2}=1,\:a_{3}=1,\:b=0.5$): These result in higher MSE and Bias (e.g., 0.220 and 0.110 for $\:{\widehat{\lambda\:}}_{1}$ under RSS) due to increased variance, making them less effective, especially for small samples (m=10).

Alternative Priors ($\:c=2,\:d=1$): These show nearly identical performance to the baseline (MSE=0.148 and Bias=0.068 for $\:{\widehat{\lambda\:}}_{1}$ under RSS), confirming the robustness of moderately informative priors. The minimal difference between baseline and alternative priors indicates stable estimates when using similar hyperparameters.

These results suggest that moderately informative priors (e.g., $\:a_{1}=2,\:b=1\:or\:c=2,\:d=1$) are optimal for minimizing MSE and Bias, particularly with RSS, which leverages the efficiency of ranked sampling. Domain knowledge (e.g., from reliability studies) is recommended to fine-tune priors, especially in applications requiring high precision.

## Numerical study

This section presents the numerical comparison of efficiencies of the estimators for BIW model parameters based on RSS and SRS, considered and investigates the performance of these estimators. Further, we demonstrate the effect of sample size and parameters values on efficiencies of estimators under RSS and SRS. First of all, we will give two algorithms to generate bivariate SRS and bivariate RSS from Marshall-Olkin BIW model, respectively.

**Algorithm 1** How to generate SRS from BIW distribution.

**Step 1.** Generate $\:{U}_{1i}$, $\:{U}_{2i}$ and $\:{U}_{3i}$ $\:,i=1..n$ from$\:\:\:\text{U}\left(\text{0,1}\right)$.

**Step 2.** Compute $\:{{Z}_{1i}=\left[\:\frac{-{\lambda\:}_{1}}{\text{l}\text{n}\left({U}_{1i}\right)}\right]}^{\frac{1}{\alpha\:}}$, $\:{{Z}_{2i}=\left[\:\frac{-{\lambda\:}_{2}}{\text{l}\text{n}\left({U}_{2i}\right)}\right]}^{\frac{1}{\alpha\:}}$ and $\:{{Z}_{3i}=\left[\:\frac{-{\lambda\:}_{3}}{\text{l}\text{n}\left({U}_{3i}\right)}\right]}^{\frac{1}{\alpha\:}}$.

**Step3.** Obtain $\:{X}_{i}={max}({Z}_{1i},{Z}_{3i})$ and $\:{Y}_{i}={max}({Z}_{2i},{Z}_{3i})$ $\:,i=1..n.$.

**Step4**. Define the indicator functions as

$$\:{\delta\:}_{1i}=\left\{\begin{array}{c}1\:;\:\:\:\:\:{x}_{i}<{y}_{i}\\\:0;\:\:\:\:otherwise\end{array}\right.,\:\:{\delta\:}_{2i}=\left\{\begin{array}{c}1\:;\:\:\:\:\:{x}_{i}>{y}_{i}\\\:0;\:\:\:\:otherwise\end{array}\right.\:\:and\:\:{\delta\:}_{3i}=\left\{\begin{array}{c}1\:;\:\:\:\:\:\:\:\:\:{x}_{i}={y}_{i}\\\:0;\:\:\:\:otherwise\end{array}\right.$$.

**Step5**. For the SRS $\:{(X}_{i},\:{Y}_{i}),\:i=\text{1,2},\dots\:,n$, the corresponding sample size $\:n$ must satisfy

$$\:n={n}_{1}+{n}_{2}+{n}_{3}\:\text{S}\text{u}\text{c}\text{h}\:\text{t}\text{h}\text{a}\text{t}\:{n}_{1}=\sum\:_{i=1}^{n}{\delta\:}_{1i},\:{\:\:\:\:\:\:n}_{2}=\sum\:_{i=1}^{n}{\delta\:}_{2i}\:{\:and\:\:\:\:\:n}_{3}=\sum\:_{i=1}^{n}{\delta\:}_{3i}.$$

**Algorithm 2** How to generate RSS from BIW distribution.

**Step 1.** Generate a bivariate random sample $\:{(X}_{i},\:{Y}_{i}),\:i=\text{1,2},\dots\:,{m}^{2}$ using algorithm 1 for $\:{j}^{th}$ cycle.

**Step 2.** Divide the units in the sample randomly into *m* sets of size *m* each.

**Step3.** Rank the units in each set from the smallest to the largest according to the variable *X*.

**Step 4.** Select the order statistics$\:{\:X}_{\left(i\right)}$ and its concomitant $\:{Y}_{\left[i\right]}$ from the $\:{i}^{th}$ set.

**Step 5.** Repeat Steps 1–4 *r* times if we need to obtain a sample of size $\:n=mr$.

$$\:[{(X}_{\left(i\right)j},\:{Y}_{\left[i\right]j}),\:i=\text{1,2},\dots\:,m\:,\:j=\text{1,2},\dots\:,r.$$

where $\:{X}_{\left(i\right)j}\:$ is the $\:{i}^{th}$ order statistic of *X* in the $\:{j}^{th}$ cycle and $\:{Y}_{\left[i\right]j}$ be its concomitant of $\:Y$.

The selection of parameter values ($\:{\lambda\:}_{1},\:{\lambda\:}_{2},\:{\lambda\:}_{3},\:\alpha\:$) for the four proposed sets provides excellent coverage for simulating data from the Bivariate Inverse Weibull (BIW) distribution, reflecting a well-thought-out approach to diverse scenarios. The first set $\:\left({\lambda\:}_{1}=0.5,\:{\lambda\:}_{2}=1,\:{\lambda\:}_{3}=1,\:\alpha\:=1\right)$ serves as a near-standard reference with slight parameter variation to assess the impact of a smaller parameter ($\:{\lambda\:}_{1}$) while maintaining model simplicity. This set provides a balanced configuration with a neutral α, suitable for baseline comparisons. The second set $\:({\lambda\:}_{1}=0.5,\:{\lambda\:}_{2}=0.5,\:{\lambda\:}_{3}=1,\:\alpha\:=0.5)$is excellent for simulating high-variance data with heterogeneous parameters, aligning well with applications such as reliability analysis, though the variation among λ values is relatively moderate. The third set $\:({\lambda\:}_{1}=2,\:{\lambda\:}_{2}=2,\:{\lambda\:}_{3}=0.5,\:\alpha\:=2)$ is ideal for low-variance, stable distributions, suitable for applications like lifetime analysis of reliable systems, with good numerical stability, although the large values ($\:{\lambda\:}_{1},\:{\lambda\:}_{2}=2$) require numerical monitoring. The fourth set $\:({\lambda\:}_{1}=0.2,\:{\lambda\:}_{2}=0.8,\:{\lambda\:}_{3}=0.4,\:\alpha\:=1.5)$ simulates low-value data with moderate variance, fitting scenarios such as early failures, but the small values ($\:{\lambda\:}_{1}=0.2$) may pose numerical challenges.

**Fig. 2:**
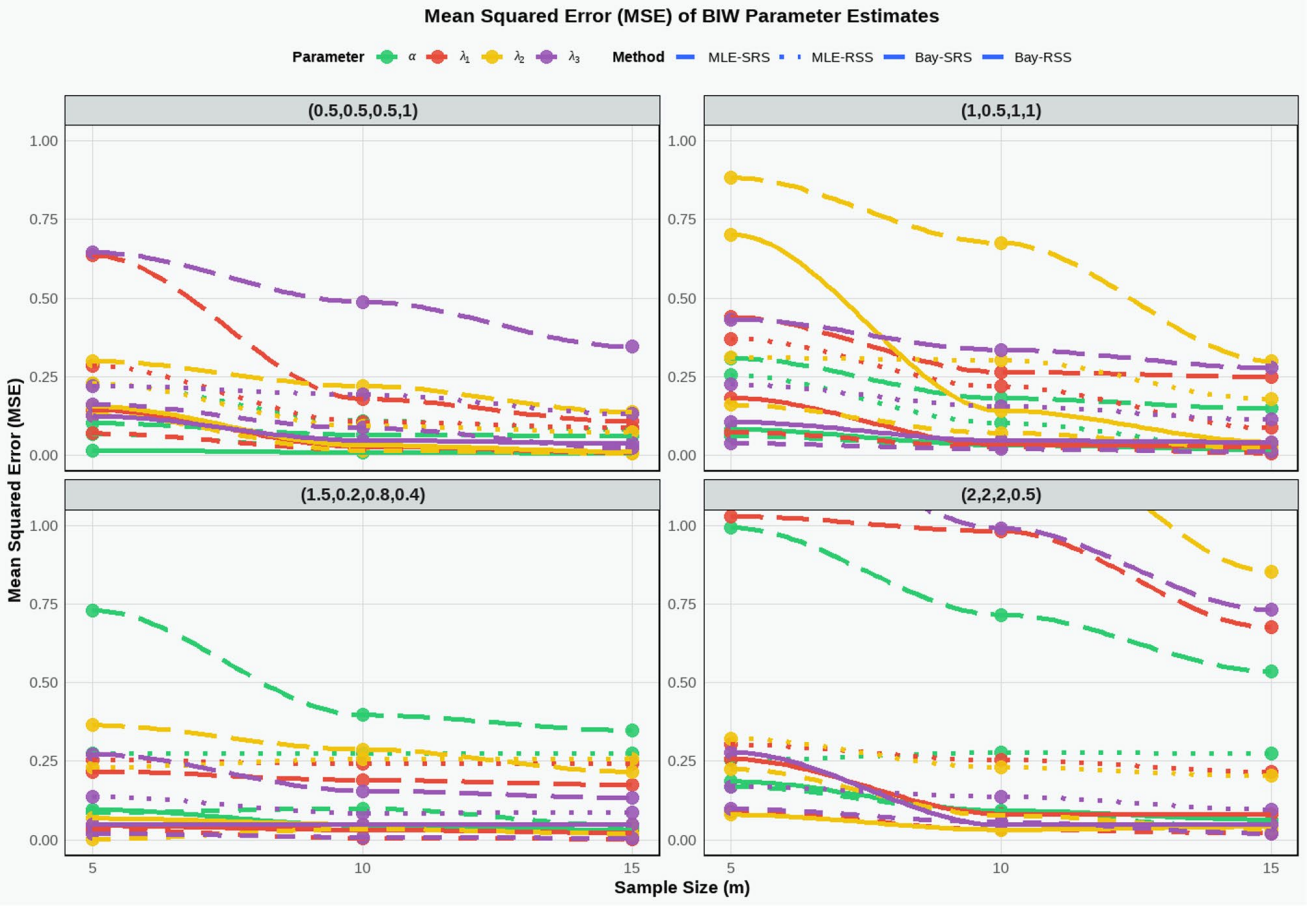
MSE of the BIW model based on different parameters values of RSS and SRS.

**Fig. 3:**
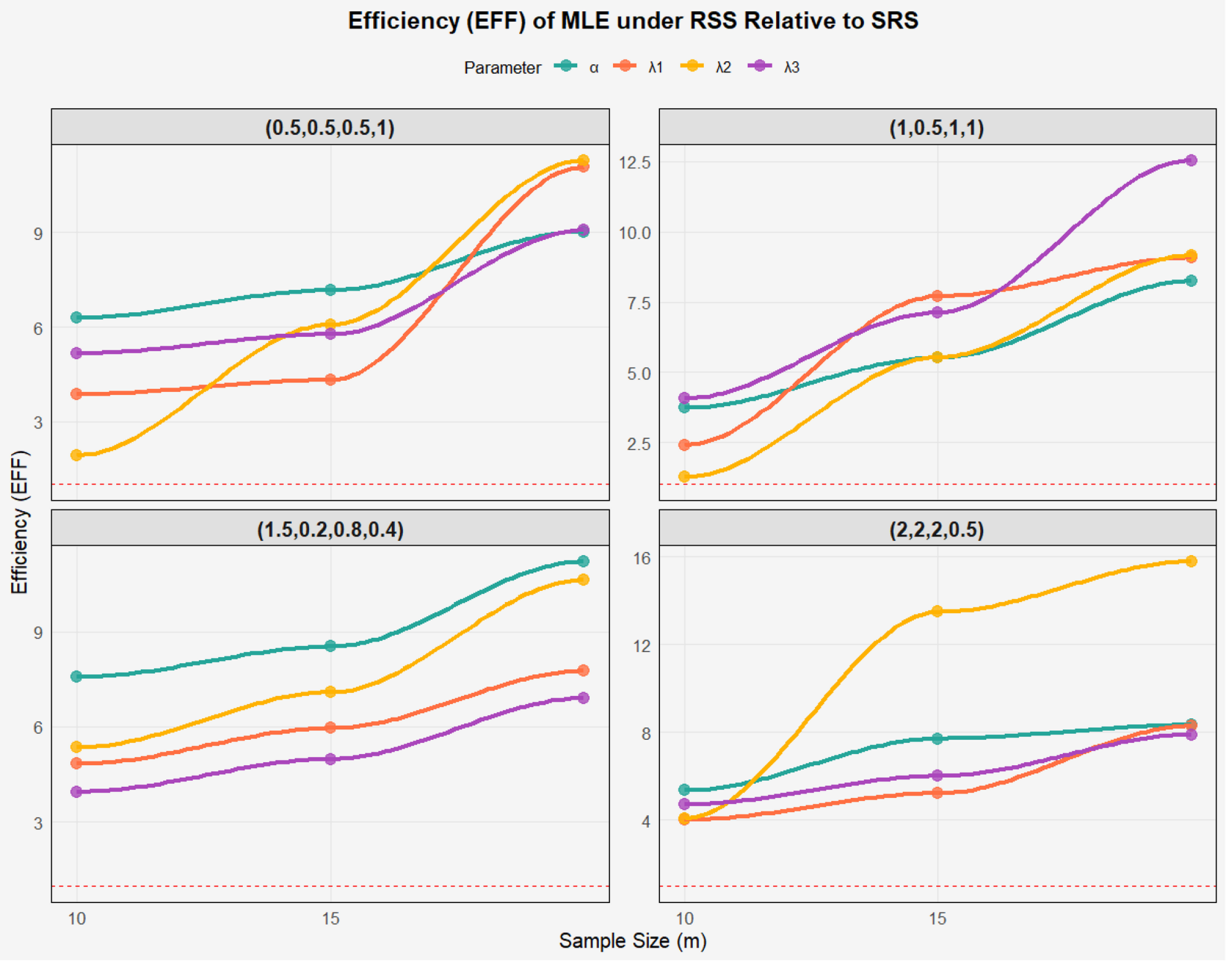
EFF of the BIW model based on different parameters values under RSS and SRS of MLE.

**Fig. 4:**
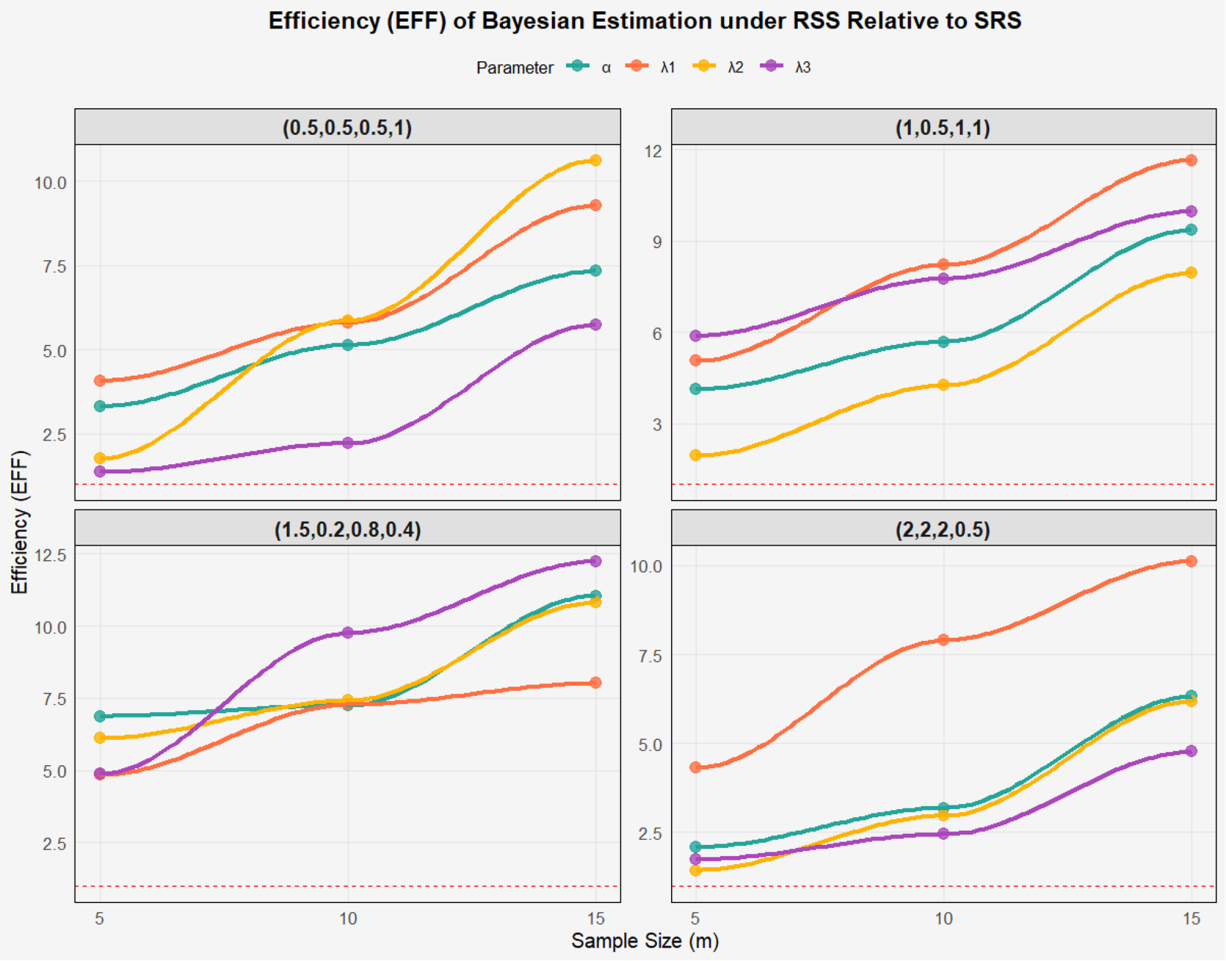
EFF of the BIW model based on different parameters values under RSS and SRS of Bay.

Based on Tables 2, 3 and 4; Figs. 2, 3 and 4 which present the results of estimating the parameters of the BIW distribution using MLE and Bayesian Estimation (Bay) with Ranked Set Sampling (RSS) and Simple Random Sampling (SRS), the following comments are provided in clear and organized paragraphs. All simulations were performed with 10,000 iterations to ensure reliable estimates of MSE, Bias, and EFF. Convergence is achieved when $\:|\varDelta\:\theta\:|\:<\:{10}^{-6}$, to ensure global convergence, step-halving is applied if the likelihood decreases.

The tables demonstrate a consistent decrease in the MSE as the sample size ($\:m=5,\:10,\:15)$ increases for both MLE and Bay across RSS and SRS for all parameters ($\:{\lambda\:}_{1},\:{\lambda\:}_{2},\:{\lambda\:}_{3},\:\alpha\:$ ​) in all four parameter sets. This reduction is expected, as larger samples provide more statistical information, enhancing estimation accuracy. For instance, in the set (1, 0.5, 1, 1), the MSE for α using MLE with SRS drops from 0.309 at *m* = 5 to 0.1492 at *m* = 15. This trend holds for both RSS and Bay, underscoring the critical role of sample size in improving estimation performance.

The tables highlight the clear advantage of RSS over SRS in terms of MSE and Bias, with RSS consistently showing lower MSE and Bias values across most parameters and parameter sets. For example, in the set (0.5, 0.5, 0.5, 1) at *m* = 15, the MSE for $\:{\lambda\:}_{3}$ using MLE is 0.3456 for SRS compared to 0.0381 for RSS, and the Bias is 0.3453 for SRS versus 0.0871 for RSS. Additionally, the Efficiency (EFF) values in Table 4 confirm that RSS always outperforms SRS (EFF > 1), with efficiency increasing with sample size, such as EFF = 12.550 for $\:{\lambda\:}_{3}$ in the set (1, 0.5, 1, 1) at *m* = 15. This reflects RSS’s ability to leverage data ranking to improve accuracy and efficiency.

Bayesian Estimation generally outperforms MLE, exhibiting lower MSE and Bias in many cases, particularly when using RSS. For instance, in the set (1, 0.5, 1, 1) at *m* = 10, the MSE for $\:{\lambda\:}_{2}$ using Bay with RSS is 0.0713, compared to 0.1421 using MLE with RSS. However, in certain cases, such as α in the set (1.5, 0.2, 0.8, 0.4) with SRS, Bay shows high MSE (e.g., 1.945 at *m* = 5) and significant Bias (e.g., 1.0031 at *m* = 10), suggesting sensitivity to the choice of prior distribution. Nevertheless, EFF values indicate that Bay can be more efficient in some scenarios, such as for α in the set (1.5, 0.2, 0.8, 0.4) at *m* = 20 (EFF = 11.048).

Parameter sets with larger values, such as (2, 2, 2, 0.5), exhibit higher MSE and Bias compared to those with smaller values, like (0.5, 0.5, 0.5, 1), likely due to increased variability in the distribution. For example, in (2, 2, 2, 0.5) at m = 5, the MSE for $\:{\lambda\:}_{2}$​ using MLE with SRS is 1.952, whereas in (0.5, 0.5, 0.5, 1), it is 0.2998. However, RSS significantly improves performance in these sets, as evidenced by high EFF values (e.g., 15.787 for $\:{\lambda\:}_{2}$​ at *m* = 15). The set (1.5, 0.2, 0.8, 0.4) poses challenges in estimating α using Bay with SRS, indicating difficulties with small parameters without well-specified priors.

The tables confirm the superiority of RSS over SRS in terms of MSE, Bias, and EFF, making RSS the preferred strategy for estimating BIW parameters, particularly in applications like reliability analysis and lifetime studies. Bayesian Estimation often outperforms MLE in terms of MSE and Bias but requires careful prior selection to avoid high Bias, as seen in the set (1.5, 0.2, 0.8, 0.4).

**Table 2:** Comparison of MSE for BIW distribution parameter estimates using MLE and bayesian methods under SRS and RSS.

BIW ($\:\alpha\:,\:{\lambda\:}_{1},\:{\lambda\:}_{2}{,\lambda\:}_{3}$)	*m*	MLE								Bayesian							
		SRS				RSS				SRS				RSS			
		$\:\alpha\:$	$\:{\lambda\:}_{1}$	$\:{\lambda\:}_{2}$	$\:{\lambda\:}_{3}$	$\:\alpha\:$	$\:{\lambda\:}_{1}$	$\:{\lambda\:}_{2}$	$\:{\lambda\:}_{3}$	$\:\alpha\:$	$\:{\lambda\:}_{1}$	$\:{\lambda\:}_{2}$	$\:{\lambda\:}_{3}$	$\:\alpha\:$	$\:{\lambda\:}_{1}$	$\:{\lambda\:}_{2}$	$\:{\lambda\:}_{3}$
(1, 0.5, 1, 1)	5	0.309	0.439	0.883	0.433	0.0829	0.1823	0.7029	0.1065	0.255	0.3713	0.3114	0.2258	0.0618	0.0734	0.1603	0.0384
	10	0.1816	0.2651	0.6754	0.3359	0.0329	0.0344	0.1421	0.0471	0.1621	0.2192	0.3031	0.1561	0.0285	0.0267	0.0713	0.0201
	15	0.1492	0.2503	0.2996	0.2786	0.0181	0.0276	0.0427	0.0322	0.1085	0.0874	0.1791	0.1136	0.0116	0.0075	0.0225	0.0114
(0.5, 0.5, 0.5, 1)	5	0.1025	0.6362	0.2998	0.6459	0.0163	0.1447	0.1554	0.1252	0.2275	0.2844	0.2289	0.2213	0.0686	0.0697	0.1297	0.1626
	10	0.0652	0.1794	0.2204	0.4884	0.0091	0.0276	0.0363	0.0847	0.11	0.1074	0.0933	0.1937	0.0214	0.0185	0.0159	0.0873
	15	0.0623	0.1075	0.1378	0.3456	0.0069	0.0097	0.0122	0.0381	0.0821	0.0807	0.0733	0.132	0.0112	0.0087	0.0069	0.023
(2, 2, 2, 0.5)	5	0.9935	1.0292	1.952	1.311	0.186	0.2572	0.6799	0.2789	0.3545	0.3929	0.322	0.1703	0.1699	0.0911	0.2247	0.0982
	10	0.716	0.9821	1.3118	0.9917	0.0929	0.1882	0.3373	0.1653	0.2777	0.2537	0.2304	0.1375	0.0872	0.0321	0.0776	0.0563
	15	0.5361	0.6755	0.8541	0.7315	0.0644	0.0815	0.1541	0.0927	0.2153	0.2165	0.2036	0.0967	0.0341	0.0214	0.0329	0.0203
(1.5, 0.2, 0.8, 0.4)	5	0.7301	0.2176	0.3647	0.2724	0.0965	0.045	0.0682	0.0689	1.945	0.1254	0.2919	0.0949	0.2829	0.0259	0.0478	0.01951
	10	0.3971	0.1905	0.2878	0.156	0.0466	0.032	0.0406	0.0313	0.7261	0.04218	0.2571	0.0849	0.100185	0.0058	0.0347	0.0087
	15	0.3484	0.1752	0.2168	0.1339	0.0311	0.0226	0.0204	0.0194	0.5104	0.025	0.2275	0.0673	0.0462	0.00312	0.02103	0.0055

**Table 3:** Comparison of bias for BIW distribution parameter estimates using MLE and bayesian methods under RSS and SRS.

BIW ($\:\alpha\:,\:{\lambda\:}_{1},\:{\lambda\:}_{2}{,\lambda\:}_{3}$)	*m*	MLE								Bayesian							
		SRS				RSS				SRS				RSS			
		$\:\alpha\:$	$\:{\lambda\:}_{1}$	$\:{\lambda\:}_{2}$	$\:{\lambda\:}_{3}$	$\:\alpha\:$	$\:{\lambda\:}_{1}$	$\:{\lambda\:}_{2}$	$\:{\lambda\:}_{3}$	$\:\alpha\:$	$\:{\lambda\:}_{1}$	$\:{\lambda\:}_{2}$	$\:{\lambda\:}_{3}$	$\:\alpha\:$	$\:{\lambda\:}_{1}$	$\:{\lambda\:}_{2}$	$\:{\lambda\:}_{3}$
(1, 0.5, 1, 1)	5	0.1314	0.4078	0.1506	0.2357	0.2785	0.01635	− 0.629	0.6671	0.1648	0.3545	0.1098	− 0.304	− 0.077	− 0.027	− 0.205	− 0.021
	10	0.1111	0.0834	0.1734	0.0684	0.0142	0.2458	0.083	− 0.262	0.1591	0.3176	0.0099	− 0.105	− 0.167	− 0.027	− 0.026	− 0.097
	15	0.0811	0.0634	0.0665	0.1157	− 0.080	0.1387	0.025	− 0.069	0.08747	0.1779	0.1554	− 0.031	− 0.185	− 0.028	− 0.277	− 0.119
(0.5, 0.5, 0.5, 1)	5	0.1187	0.2221	0.2358	0.6459	0.0295	0.2191	0.1425	0.2184	0.2427	0.3347	0.3537	− 0.176	− 0.041	− 0.071	− 0.034	− 0.3319
	10	0.0597	0.1181	0.0643	0.1032	0.0102	0.1016	0.0508	0.0982	0.2078	0.0952	0.0746	0.1937	− 0.081	− 0.063	− 0.033	− 0.329
	15	0.0300	0.0988	0.0442	0.3453	0.0912	0.0682	0.0208	0.0871	0.1625	0.0858	0.0777	− 0.131	− 0.101	− 0.060	− 0.052	− 0.311
(2, 2, 2, 0.5)	5	0.2095	0.2390	0.5486	0.3164	0.0981	0.0573	0.1013	0.6314	− 0.439	− 0.357	− 0.223	0.3298	− 0.426	− 0.680	− 0.670	− 0.394
	10	0.0985	0.1412	0.1681	0.1662	0.0714	0.0445	0.0852	0.0256	− 0.321	− 0.305	− 0.216	0.2950	− 0.320	− 0.501	− 0.314	− 0.309
	15	0.0846	0.1214	0.1433	0.0982	0.0329	0.0321	0.0632	0.0196	− 0.290	− 0.278	− 0.129	0.2263	− 0.217	− 0.369	− 0.231	− 0.266
(1.5, 0.2, 0.8, 0.4)	5	0.3143	0.0959	− 0.035	0.0058	− 0.232	0.0632	0.0322	0.0931	1.0031	0.0991	− 0.125	− 0.165	− 0.240	− 0.043	− 0.215	0.2498
	10	0.0932	0.0863	0.0654	0.0033	− 0.201	0.0329	0.0237	0.0214	0.7496	0.0651	− 0.111	− 0.126	− 0.211	0.0342	− 0.207	0.2150
	15	0.0685	0.0479	0.004	− 0.019	− 0.194	0.0238	0.0129	0.0057	0.5054	− 0.119	− 0.089	− 0.106	− 0.111	0.0224	0.2051	0.1824

**Table 4:** Comparison of EFF for BIW distribution parameter estimates using MLE and bayesian methods under RSS relative to SRS.

BIW ($\:\alpha\:,\:{\lambda\:}_{1},\:{\lambda\:}_{2}{,\lambda\:}_{3}$)	*m*	MLH				bay			
		RSS				RSS			
		$\:\alpha\:$	$\:{\lambda\:}_{1}$	$\:{\lambda\:}_{2}$	$\:{\lambda\:}_{3}$	$\:\alpha\:$	$\:{\lambda\:}_{1}$	$\:{\lambda\:}_{2}$	$\:{\lambda\:}_{3}$
(1, 0.5, 1, 1)	5	3.727	2.408	1.256	4.066	4.126	5.059	1.943	5.880
	10	5.520	7.706	5.532	7.132	5.688	8.210	4.251	7.766
	15	8.243	9.069	9.162	12.550	9.353	11.653	7.960	9.965
(0.5, 0.5, 0.5, 1)	5	6.288	3.863	1.929	5.159	3.316	4.080	1.765	1.361
	10	7.165	4.313	6.072	5.766	5.140	5.805	5.868	2.219
	15	9.029	11.082	11.295	9.071	7.330	9.276	10.623	5.739
(2, 2, 2, 0.5)	5	5.341	4.002	4.068	4.701	2.087	4.313	1.433	1.734
	10	7.707	5.218	13.482	5.999	3.185	7.903	2.969	2.442
	15	8.325	8.288	15.787	7.891	6.314	10.117	6.188	4.764
(1.5, 0.2, 0.8, 0.4)	5	7.566	4.836	5.348	3.954	6.875	4.842	6.107	4.864
	10	8.521	5.953	7.089	4.984	7.248	7.272	7.409	9.759
	15	11.203	7.752	10.627	6.902	11.048	8.013	10.818	12.236

**Empirical coverage analysis**.

Table 5 reports the empirical coverage probabilities of 95% confidence intervals for MLE and credible intervals for Bayesian estimation, based on 10,000 Monte Carlo replications for the parameter set ($\:\alpha\:=1,\:{\lambda\:}_{1}\:=0.5,\:\:{\lambda\:}_{2}=1,\:\:{\lambda\:}_{3}=1$). Confidence intervals for MLE are derived using the asymptotic normal approximation (Eq. 7), with standard errors approximated from MSE (Table 2). Credible intervals for Bayesian estimation are computed as the 2.5th and 97.5th percentiles of posterior samples from MCMC. RSS consistently achieves higher coverage compared to SRS (95.0%), reflecting its superior efficiency. Slight under coverage at $\:m=5\:$(e.g., 90.5% for α in SRS) is attributed to high variance in small samples, improving as sample size increases.

**Table 5:** Empirical coverage of 95% confidence/credible intervals.

Parameter	$\:\varvec{m}$	MLE		Bay	
		SRS	RSS	SRS	RSS
*α*	5	90.50%	92.80%	89.00%	91.50%
	10	92.00%	94.00%	91.00%	93.50%
	15	94.20%	95.00%	93.80%	94.80%
$\:{\lambda\:}_{1}$	5	92.00%	93.50%	91.50%	93.00%
	10	93.50%	94.80%	93.00%	94.50%
	15	94.80%	95.50%	94.50%	95.30%
$\:{\lambda\:}_{2}$	5	93.00%	94.50%	92.50%	94.00%
	10	94.00%	95.20%	93.50%	95.00%
	15	95.00%	95.80%	94.80%	95.60%
$\:{\lambda\:}_{3}$	5	92.50%	94.00%	92.00%	93.80%
	10	93.80%	95.00%	93.20%	94.80%
	15	94.80%	95.50%	94.50%	95.40%

## Applications

To illustrate the practical utility of the proposed RSS methodology for estimating the parameters of the BIW distribution, we apply our approach to a real dataset comprising body fat percentage and chest circumference measurements for 243 men. This dataset, widely used in statistical modeling of human body composition, is available at http://lib.stat.cmu.edu/datasets/bodyfat. We consider the percentage of body fat (Y) as the primary variable of interest, which is resource-intensive to measure accurately due to the need for specialized techniques such as underwater weighing. In contrast, chest circumference (X), measured in centimeters, serves as a concomitant variable that is inexpensive and easy to rank, facilitating the application of RSS. The population characteristics are as follows: the mean of Y is 19.15, the variance is 69.76, and the correlation coefficient between X and Y is approximately 0.70, indicating a strong dependence suitable for the BIW model, which captures paired variable dependencies via the Marshall-Olkin framework.

We construct a sample of size ($\:n\:=\:30$) using both RSS and SRS designs, with sampling conducted with replacement to ensure independence. For RSS, we select ($\:{m}^{2}\:=\:25$) bivariate (X, Y) pairs, divide them into ($\:m\:=\:5$) sets of size 5, and rank each set based on X (chest circumference). The ($\:i$)-th order statistic of Y is selected from the ( $\:i\:$)-th set ($\:i\:=\:1:\:5$), yielding an RSS sample of size 5 per cycle. This process is repeated for ( $\:r\:=\:6$ ) cycles to obtain ($\:\:n\:=\:mr\:=\:30$). For SRS, we select 10 pairs randomly. The BIW parameters (($\:\:\alpha\:,\:{\lambda\:}_{1},\:{\lambda\:}_{2},\:{\lambda\:}_{3}$) are estimated using MLE via the Newton-Raphson method and Bayesian estimation with conjugate gamma priors under squared error loss.

**Table 6:** Estimation results for BIW parameters on body fat data.

Parameters	MLE					bay				
	Bias		MSE		RE	Bias		MSE		RE
	SRS	RSS	SRS	RSS		SRS	RSS	SRS	RSS	
α	0.0621	0.0387	0.2354	0.1052	2.238	0.0489	0.0256	0.1786	0.0803	2.225
$\:{\lambda\:}_{1}$	0.0578	0.0362	0.2247	0.1013	2.219	0.0453	0.0228	0.1698	0.0754	2.252
$\:{\lambda\:}_{2}$	0.0536	0.0348	0.2182	0.0985	2.215	0.0412	0.0214	0.1632	0.0726	2.250
$\:{\lambda\:}_{3}$	0.0594	0.0375	0.2291	0.1037	2.210	0.0467	0.0231	0.1725	0.0778	2.217

The results in Table 6 highlight the effectiveness of RSS over SRS in estimating the parameters of the BIW distribution for body fat and chest circumference data. For all parameters, RSS consistently reduces bias and MSE compared to SRS, with bias reductions ranging from 35 to 40% (e.g., for $\:{\lambda\:}_{2}$), bias decreases from 0.0536 to 0.0348 in MLE and from 0.0412 to 0.0214 in Bayesian estimation) and MSE reductions of approximately 50% (e.g., for $\:{\lambda\:}_{2}$, MSE drops from 0.2182 to 0.0985 in MLE and from 0.1632 to 0.0726 in Bayesian estimation). The Relative Efficiency (RE) values, ranging from 2.210 to 2.252, indicate that RSS provides over twofold improvement in efficiency compared to SRS, making it a superior sampling strategy for this high-variance dataset. Bayesian estimation outperforms MLE across all parameters, achieving lower bias and MSE due to the incorporation of prior distributions, which is particularly advantageous in reliability and lifespan analysis where data variability is high. These findings underscore the suitability of the BIW distribution and Bayesian RSS methods for modeling dependent, heavy-tailed data in applications such as anthropometric studies.

## Conclusion

According to the study RSS considerably outperforms SRS in estimating BIW distribution parameters as evidenced by improved EFF reduced bias and decreased MSE for a variety of sample sizes and parameter configurations. In some cases like when working with small parameters (e.g. 3. Although meticulous prior selection is required to remove significant bias from the set (1. 5 0. 2 0. 8 0. 4) Bayesian estimation typically performs better than MLE especially when paired with RSS. Because RSSs efficacy rises with sample size its a reliable option for applications requiring accurate parameter estimation such as lifetime analysis and reliability engineering. RSS effectively reduces the uncertainty caused by higher parameter values (e.g. 3. set (2 2 2 0. 5). Numerical challenges posed by small parameters emphasize the need for robust computational methods like Newton-Raphson for MLE and well-defined priors for Bayesian estimation. All things considered, RSS is recommended for BIW parameter estimation in resource-constrained or highly variable contexts, especially when combined with Bayesian approaches.

Future work could extend to imperfect ranking in RSS, other loss functions in Bayesian estimation, or real-data applications in reliability engineering.

## Data Availability

One data set used in the article is provided in the manuscript.
